# Design and synthesis of chalcone derivatives as potential non-purine xanthine oxidase inhibitors

**DOI:** 10.1186/s40064-016-3485-6

**Published:** 2016-10-13

**Authors:** Trung Huu Bui, Nhan Trung Nguyen, Phu Hoang Dang, Hai Xuan Nguyen, Mai Thanh Thi Nguyen

**Affiliations:** 1 Faculty of Chemistry, University of Science, Vietnam National University, 227 Nguyen Van Cu, District 5, Ho Chi Minh City, Vietnam; 2 Cancer Research Laboratory, Vietnam National University, 227 Nguyen Van Cu, District 5, Ho Chi Minh City, Vietnam

**Keywords:** Sappanchalcone, Chalcone, Non-purine xanthine oxidase inhibitors

## Abstract

**Background:**

Based on some previous research, the chalcone derivatives exhibited potent xanthine oxidase inhibitory activity, e.g. sappanchalcone (**7**), with IC_50_ value of 3.9 μM, was isolated from *Caesalpinia sappan*. Therefore, objectives of this research are design and synthesis of **7** and other chalcone derivatives by Claisen–Schmidt condensation and then evaluate their XO inhibitory activity.

**Results:**

Fifteen chalcone derivatives were synthesized by Claisen–Schmidt condensation, and were evaluated for XO inhibitory activity. Nine out of 15 synthetic chalcones showed inhibitory activity (**3**; **5**–**8**; **10**–**13**). Sappanchalcone derivatives (**11**) (IC_50_, 2.5 μM) and a novel chalcone (**13**) (IC_50_, 2.4 μM) displayed strong xanthine oxidase inhibitory activity that is comparable to allopurinol (IC_50_, 2.5 μM). The structure–activity relationship of these chalcone derivatives was also presented.

**Conclusions:**

It is the first research on synthesis sappanchalcone (**7**) by Claisen–Schmidt condensation. The overall yield of this procedure was 6.6 %, higher than that of reported procedure (4 %). Design, synthesis, and evaluation of chalcone derivatives were carried out. This result suggests that the chalcone derivative can be used as potential non-purine XO inhibitors.

**Graphical abstract:**
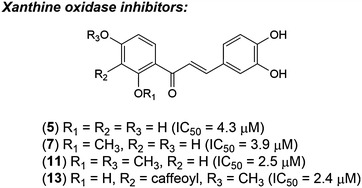
The chalcone derivatives as potential non-purine xanthine oxidase inhibitors

**Electronic supplementary material:**

The online version of this article (doi:10.1186/s40064-016-3485-6) contains supplementary material, which is available to authorized users.

## Background

Xanthine oxidase (XO) is a key enzyme in purine metabolic pathway. This complex metalloflavoprotein catalyzes the oxidation of hypoxanthine into xanthine and then finally into uric acid (Massey et al. 1969). Overproduction or under excretion of uric acid leads to hyperuricemia, a key cause of gout (Scott and Agudelo 2003). Also, hyperuricemia has been identified as an independent risk factor for chronic kidney and cardiovascular diseases (Edwards 2008; Nakagawa et al. 2006); thus, maintaining uric acid at a normal level is an important therapy to prevent gout. In many kinds of research, XO has been targeted as a promising agent for treatment of hyperuricemia. Allopurinol is a potent XO inhibitor with a purine backbone and has been used clinically for more than 40 years (Murata et al. 2009). Unfortunately, this drug has infrequent and severe side effects as in the cause of hypersensitivity syndrome (Hammer et al. 2001), Stevens–Johnson syndrome (Fritsch and Sidoroff 2000), and renal toxicity (Horiuchi et al. 2000). Therefore, there is a need to develop other novel chemical structural types of XO inhibitors.

Chalcones are within a class of chemical compounds that widely exist in a variety of medicinal plants. Claisen–Schmidt condensation, a base catalyzed condensation, was found to be most convenient to synthesize chalcones. Their flexible structure allows them to possess a large number of biological activities including antitumor, antifungal, antiprotozoal, antimitotic, and antiviral (Zhang et al. 2013). Some chalcone derivatives exhibited potent XO inhibitory activity (Beiler and Martin 1951; Niu et al. 2011).

Our preliminary screening to search for XO inhibitory activity of Vietnamese medicinal plants revealed that the methanolic extract of *Caesalpinia sappan’s* heartwood exhibited significant XO inhibitory activity with an IC_50_ value of 14.2 μg/mL (Nguyen et al. 2005). The bioactivity-guided fractionation of MeOH extract of *C. sappan*’s heartwood was carried out. Sappanchalcone (**7**) was isolated from EtOAc-soluble fraction (IC_50_, 12.8 μg/mL); this compound displayed the most potent activity with an IC_50_ value of 3.9 μM, comparable to that of allopurinol (IC_50_, 2.5 μM) (Nguyen et al. 2005). To study the possibility of using **7** as gout treatment required a large amount of this compound but the amount of **7** in *C. sappan* is very low.

The synthesis of **7** was carried out by Heck coupling reaction followed by demethylation (Bianco et al. 2004). Therefore, objectives of this research are design and synthesis of **7** and other chalcone derivatives by Claisen–Schmidt condensation and then evaluate their XO inhibitory activity.

## Results and discussion

**Scheme 1:**
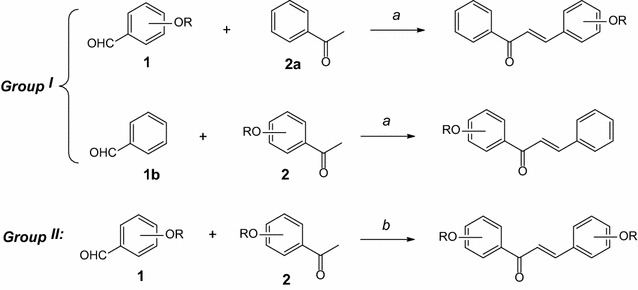
Synthesis of chalcones in group I and group II. Reagents and conditions: *a* KOH_aq_, MeOH, ultrasound-assisted; *b* KOH_aq_, ultrasound-assisted

As outlined in Scheme 1, some known and novel chalcone analogs (group I: the hydroxyl groups attached to one of two aromatic rings of chalcones; and group II: both two aromatic rings carried the hydroxy groups) were prepared via Claisen Schmidt condensation reactions between appropriate benzaldehydes and aryl methyl ketones. The reaction was monitored by thin-layer chromatography (TLC). The reaction mixture after aldol condensation was acidified and cooled to obtain the crude product. Pure chalcone was purified by recrystallization and structure elucidation was determined by NMR spectroscopy. The overall yield of the reaction was then measured by HPLC–UV/260 nm.

For the purpose of simplifying the synthesis, the protecting group was not carried out, so the concentration of aqueous alkaline base was critical in Claisen–Schmidt condensation. Therefore, typical reactions affording 3,4-dihydroxychalcone (**3**) and 3,4,2ʹ,4ʹ-tetrahydroxychalcone (**5**) were investigated in the presence of different concentrations of the aqueous solution of KOH at room temperature 30 °C (Table 1).

**Table 1:** Optimal condition for the concentration of KOH

Entry	C_KOH_ (M)	Yield (%)
1^a^	6	28.3
2^a^	8	31.1
3^a^	10	39.7
4^a^	12	37.8
5^a^	14	34.4
6^b^	10	20.5
7^b^	11	24.5
8^b^	12	26.8
9^b^	13	28.4
10^b^	14	33.4
11^b^	−^d^	32.8
12^c^	10	8.3
13^c^	11	17.5
14^c^	12	22.8
15^c^	13	7.1

^a^Synthesis of chalcone in group I: **1a/2a** = 1/1; MeOH (1.00 mL); 30 °C; UA; 6 h

^b^Synthesis of chalcone in group II: **1a/2b** = 1/1; H_2_O (1.00 mL); 30 °C; UA; 6 h

^c^Synthesis of **7**: **1a/2c** = 2/1; H_2_O (1.00 mL); 80 °C; UA; 8 h

^d^Using a solid KOH

The synthesis of **3**, in the presence of 1.00 mL of MeOH as the solvent, and aqueous base with different concentrations from 6 to 14 M, together with ultrasound-assisted (UA), afforded the highest yield of **3** (39.7 %) when the reaction was carried out at KOH 10 M (Table 1, entry 3). When comparing the synthesis of **5** and that of **3**, both were synthesized from 3,4-dihydroxybenzaldehyde (**1a**), differed only in acetophenone derivatives. In this case, we used 2ʹ,4ʹ-dihydroxyacetophenone (**2b**), a more polar substrate than acetophenone (**2a**). The use of MeOH solvent was not necessary because both substrates were dissolved in alkaline solution well; and highest yield of **5** (33.4 %) was afforded when KOH 14 M (Table 1, entry 10) was used.

From results in Tables 2 and 3, the yield of these typical reactions increased up to a period and then stopped changing. The reaction time may vary depending on different activation methods i.e. conventional heating (entry 1–4 in Tables 2 or 3) or ultrasound-assisted (entry 5–11 in Tables 2 or 3). The reaction temperature was significantly impacted yield of the synthesis of **3** and **5**; it can be seen that the optimal reaction temperatures were 70 °C (Table 2, entry 13) and 80 °C (Table 3, entry 14), respectively. Due to limited solubility in the aqueous base of acetophenone (**2a**), using a suitable organic solvent and appropriate volume is crucial to synthesize **3**. Therefore, under these optimal conditions, an investigation on the effect of volume of MeOH (Table 2, entry 13, 16–19) was carried out.

**Table 2:** Optimization of parameters for the synthesis of 3

Entry	Temp. (^o^C)	Time (hours)	Molar ratio (1a/2a)	Volume of MeOH (mL)	Yield (%)
1^a^	30	6	1:1	1.00	24.1
2^a^	30	12	1:1	1.00	29.2
3^a^	30	18	1:1	1.00	36.2
4^a^	30	24	1:1	1.00	38.3
5^b^	30	2	1:1	1.00	24.7
6^b^	30	3	1:1	1.00	27.5
7^b^	30	4	1:1	1.00	29.0
8^b^	30	5	1:1	1.00	31.9
9^b^	30	6	1:1	1.00	38.0
10^b^	30	7	1:1	1.00	36.8
11^b^	30	8	1:1	1.00	36.1
12^b^	60	6	1:1	1.00	46.3
13^b^	70	6	1:1	1.00	52.1
14^b^	80	6	1:1	1.00	47.7
15^b^	90	6	1:1	1.00	45.1
16^b^	70	6	1:1	0.25	32.2
17^b^	70	6	1:1	0.50	40.8
18^b^	70	6	1:1	0.75	46.5
19^b^	70	6	1:1	1.50	51.5
20^b^	70	6	1.5:1	1.00	70.6
21^b^	70	6	2:1	1.00	77.3
22^b^	70	6	2.5:1	1.00	79.8
23^b^	70	6	3:1	1.00	81.6

Reaction was carried out at KOH 10 M

^a^Using CH

^b^Using UA

**Table 3:** Optimization of parameters for the synthesis of 5

Entry	Temp. (°C)	Time (h)	Molar ratio (1a/2b)	Yield (%)
1^a^	30	12	1:1	24.1
2^a^	30	24	1:1	29.2
3^a^	30	36	1:1	36.2
4^a^	30	48	1:1	38.3
5^b^	30	4	1:1	23.8
6^b^	30	5	1:1	28.4
7^b^	30	6	1:1	32.7
8^b^	30	7	1:1	36.9
9^b^	30	8	1:1	44.0
10^b^	30	10	1:1	40.8
11^b^	30	12	1:1	38.1
12^b^	60	8	1:1	47.1
13^b^	70	8	1:1	50.9
14^b^	80	8	1:1	57.1
15^b^	90	8	1:1	55.4
16^b^	80	8	1.5:1	64.3
17^b^	80	8	2:1	69.8
18^b^	80	8	2.5:1	54.2
19^b^	80	8	3:1	47.6

Reaction was carried out at KOH 14 M

^a^Using CH

^b^Using UA

The molar ratio of two reactants (**1a**/**2a** or **1a**/**2b**) was also investigated (Table 2, entry 13, 20–23; and Table 3, entry 14, 16–19). When the molar ratio of benzaldehyde and acetophenone derivatives was 2.5:1 or 3:1, the residual reactants and desired products crystallized simultaneously. So, the recrystallization was not be used to purify the crude product. Therefore, the molar ratio of two reactants of 2:1 was recommended in our case. From the above results, a set of conditions to synthesize the chalcone in group I [3,4-dihydroxychalcone (**3**) and 2′,4′-dihydroxychalcone (**4**)] was proposed: reaction was carried out at KOH 10 M, under ultrasound-assisted for 6 h at 70 °C, using 1.00 mL of MeOH as solvent and molar ratio of **1**/**2** = 2:1 (Table 2, entry 21). Moreover, that of the chalcone in group II [3,4,2ʹ,4ʹ-tetrahydroxychalcone (**5**) and 2,4,2′,4′-tetrahydroxychalcone (**6**)], as follows: reaction was carried out at KOH 14 M, under ultrasound-assisted for 8 h at 80 °C, and molar ratio of **1**/**2** = 2: 1 (Table 3, entry 17).

**Scheme 2:**
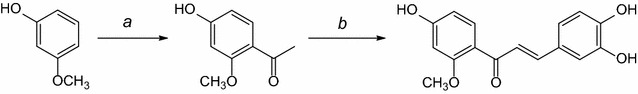
Synthesis of sappanchalcone (**7**). Reagent and conditions: *a* CH_3_COOH, polyphosphoric acid, 60 °C, 30 min; *b* 2ʹ,4ʹ-dihydroxyacetophenone, KOH 12 M, ultrasound-assisted, 80 °C, 8 h

Sappanchalcone (**7**) was synthesized by the reaction of 4ʹ-hydroxy-2ʹ-methoxyacetophenone (**2c**) with 3,4-dihydroxybenzaldehyde (**1a**) (Scheme 2). However, 4ʹ-hydroxy-2ʹ-methoxyacetophenone (**2c**) has not been yet widely commercialized. It was synthesized by the acetylation of 3-methoxyphenol and acetic acid in the presence of polyphosphoric acid (P_2_O_5_ > 85 %) as a catalyst (Nagai et al. 1984; Nakazawa 1954). However, this reaction also obtained two other by-products with significant yield: 2ʹ-hydroxy-4ʹ-methoxyacetophenone (**2d**) and 3ʹ-acetyl-2ʹ-hydroxy-4ʹ-methoxyacetophenone (**2e**).

Compound **7**, both two aromatic rings carried the hydroxy groups, so it was classified as group II. However, with above optimal conditions, the desired product was not observed. In compound **2c**, the methoxyl group at position C(2ʹ) was less polar than hydroxyl group, then changed the reactivity of compound **2c** comparing to compound **2b**. Therefore, the KOH concentration was again investigated while other optimal parameters have remained the same as in the synthesis of chalcone in group II (Table 1, entry 12–15).

**Scheme 3:**
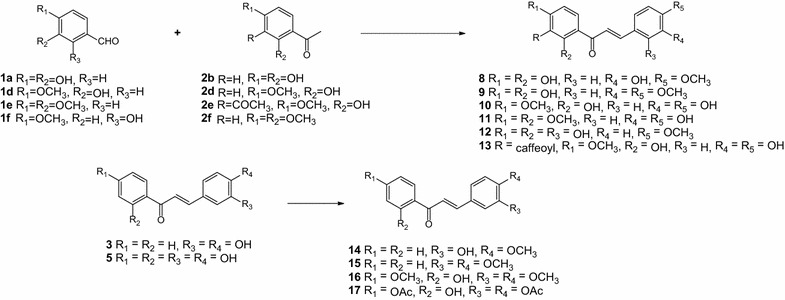
Synthesis of chalcone derivatives (**8**–**17**)

Bioactivity of chalcone depended largely on amount and properties of substituents on two phenyl rings. Especially the hydroxyl groups were considered as key substituents that significantly enhance the activity of chalcone derivatives. Therefore, we carried out the O-methylation and O-acetylation reactions of some reactants and chalcones, to diversify the chalcone derivatives. For this purpose, (1) the O-methylation reaction on three substrates: 3,4-dihydroxybenzaldehyde (**1a**), 2,4-dihydroxybenzaldehyde (**1c**) and 2ʹ,4ʹ-dihydroxyacetophenone (**2b**); (2) the O-methylation reaction on two products: 3,4-dihydroxychalcone (**3**) and 3,4,2ʹ,4ʹ-tetrahydroxychalcone (**5**); and (3) the O-acetylation reaction on 3,4,2ʹ,4ʹ-tetrahydroxychalcone (**5**) were carried out. With these schemes, ten chalcone derivatives: 3,2ʹ,4ʹ-trihydroxy-4-methoxychalcone (**8**); 2ʹ,4ʹ-dihydroxy-3,4-dimethoxychalcone (**9**); 3,4,2ʹ-trihydroxy-4ʹ-methoxychalcone (**10**); 3,4-dihydroxy-2ʹ,4ʹ-dimethoxychalcone (**11**); 2,2ʹ,4ʹ-trihydroxy-4-methoxychalcone (**12**); 3ʹ-caffeoyl-3,4,2ʹ-trihydroxy-4ʹ-methoxychalcone (**13**); 3-hydroxy-4-methoxychalcone (**14**); 3,4-dimethoxychalcone (**15**); 2ʹ-hydroxy-3,4,4ʹ-trimethoxychalcone (**16**); and 3,4,4ʹ-triacetoxy-2ʹ-hydroxychalcone (**17**) were obtained (Scheme 3). NMR data validated the formation of these chalcones (Additional file 1). Moreover, two novel chalcones (**13** and **17**) were also identified by HRMS data (Additional file 1).

XO inhibitory activity of the synthetic chalcone derivatives (**3**–**17**) and purchased chalcone (**18**) was examined by using allopurinol as a positive control. Among fifteen synthetic chalcones, nine compounds showed XO inhibitory activity with IC_50_ values <50 μM (Table 4). Four of these compounds displayed potent activity (**5**, **7**, **11** and **13** with IC_50_ values ranging from 2.4 to 4.3 μM), comparing to positive control, allopurinol (IC_50_, 2.5 μM). Compounds **6**, **10** and **12** showed relatively strong inhibitory activity with IC_50_, 16.3, 19.2 and 21.8 μM, respectively. Compounds **3** and **8** displayed average activity with IC_50_, 36.7 and 40.9 μM, respectively. Therefore, XO inhibitory activity of the chalcone derivatives depended on the location and number of the substituents on two phenyl rings.

**Table 4:** Chemical structure of the chalcone derivatives and their XO inhibitory activity

Compound	R_1_	R_2_	R_3_	R_4_	R_5_	R_6_	IC_50_ (μM)
**3**	H	H	H	H	OH	OH	40.9
**4**	OH	H	OH	H	H	H	>100
**5**	OH	H	OH	H	OH	OH	4.3
**6**	OH	H	OH	OH	H	OH	16.3
**7**	OMe	H	OH	H	OH	OH	3.9
**8**	OH	H	OH	H	OH	OMe	36.7
**9**	OH	H	OH	H	OMe	OMe	>100
**10**	OH	H	OMe	H	OH	OH	19.2
**11**	OMe	H	OMe	H	OH	OH	2.5
**12**	OH	H	OH	OH	H	OMe	21.8
**13**	OH	Caffeoyl	OMe	H	OH	OH	2.4
**14**	H	H	H	H	OH	OMe	>100
**15**	H	H	H	H	OMe	OMe	>100
**16**	OH	H	OMe	H	OMe	OMe	>100
**17**	OH	H	OAc	H	OAc	OAc	>100
**18**	H	H	H	H	H	H	>100
**Allopurinol**							2.5

Consequently, according to the above results, the structure–activity relationship of some synthetic chalcone derivatives (compound **3**–**18**) was evaluated. In all cases, the carbonyl group plays a major role in the XO inhibition activity of these compounds; it acts as a reactive oxygen species acceptor (Ponce et al. 2000). Likewise, the presence of hydroxyl groups composes another important bioactive region. That are mainly involved in dispersion interactions with an aromatic aminoacidic residue of the enzyme (Costantino et al. 1996). So, the activity of chalcones increases with increasing numbers of hydroxyls. The tetrahydroxychalcones (**5**, **6**) are more active than either of the dihydroxychalcones (**3**, **4**); and the non-substituted chalcone (**18**) was not displayed xanthine oxidase inhibitory activity. Moreover, the presence of hydroxyl groups at C(2′), C(4′), and C(4) plays an important role in the inhibition of XO (**5** > **6** ≫ **4**), these hydroxyl groups increase the activity through an increment in the stabilization of the aromatic ring due to inductive effect (Ponce et al. 2000). So, the methylation or acetylation of the hydroxyl groups generally decreases the inhibition activity (**3** > **14** ≈ **15**; **5** > **10** > **8** > **9** ≈ **16**; **6** > **12**; **5** ≫ **17**); the replacement of all hydroxyl groups in ring B (**9**, **15**‒**17**) has an extreme reducing effect on inhibitory activity.

The presence of hydroxyl group at C(2′) may allow ring closure in solution, thus reducing the effective concentration of the compound in its chalcone form (Beiler and Martin 1951). Thus, the methylation of C(2′) hydroxyl group causes an increase in activity (**7** > **5**, **11** > **10**). However, the presence of methoxyl groups at both C(2′) and C(4′) increases the activity (**11** > **7** > **5** > **10**) due to the activation of the keto group by oxygens on ring A (Beiler and Martin 1951).

Chalcones with no hydroxyl group in ring B (**4**) do not show activity. Moreover, these with two hydroxyl groups located at *ortho*- position on ring B at C(3) and C(4), showed stronger activity than those with the equivalent substitutes but located at the *meta*- position (**5** > **6** ≫ **4**). It is explained based on molar refractivity parameter; the high polarizability will enhance the attractive dispersion interactions with an aromatic residue of enzyme binding site through π–π stacking interactions (Costantino et al. 1996; Mathew et al. 2015). However, when the C(4) hydroxyl was methylated, the above conclusion is reversed. That may be because the methoxyl group has a positive inductive effect, while the hydroxyl ones have a negative inductive effect.

Compound **13**, a dimer-like compound of **10**, showed the most potent active due to additional a carbonyl and a catechol group.

## Methods

### General

All reagents were obtained at highest quality from commercially available sources and were used as received. All compounds were elucidated by NMR and HRMS data. Anal. TLC: aluminum plates precoated with *Merck Silica gel 60 F*_*254*_ as an adsorbent; visualization on TLC plates was done with UV light. Column chromatography (CC): silica gel (SiO_2_; *Kieselgel 40*, 0.063–0.200 mm, Merck). HPLC: *Agilent 1100 series* coupled to IR/UV/VIS detector; a *ZORBAX Eclipse Plus C18* column (particle size 5 μm, 250 × 4.6 mm i.d.); the mobile phase, MeOH/H_2_O/CH_3_COOH; flow rate, 0.5–1 mL min^−1^; the chromatograms monitored at 260 nm. Ultrasonic bath: *Branson 1210E*-*MT* ultrasonic bath, operating at 47 kHz. NMR Spectra: *NMR Bruker Avance II 500* spectrometer (at 500 and 125 MHz for ^1^H and ^13^C, resp.), at 25 °C; *δ* in ppm, *J* in Hz; HR-ESI–MS: *Bruker Daltonics micrOTOF*-*QII*; in *m/z*.

### General procedure for the synthesis of chalcones in group I (compounds **3** and **4**)

~2.0 mmol of benzaldehyde derivatives [276.2 mg of 3,4-dihydroxybenzaldehyde (**1a**); 106.1 mg of benzaldehyde (**1b**)] and ~1.0 mmol of acetophenone derivatives [120.2 mg of acetophenone (**2a**); 152.2 mg of 2ʹ,4ʹ-dihydroxyacetophenone (**2b**)] were dissolved in 1.00 mL MeOH, then 1.00 mL KOH 10 M was added. The flask containing the resulting mixture was suspended in the ultrasonic water bath at 70 °C for 6 h.

### General procedure for the synthesis of chalcones in group II (compounds **5** and **6**)

~2.0 mmol of benzaldehyde derivatives [275.9 mg of 3,4-dihydroxybenzaldehyde (**1a**); 276.3 mg of 2,4-dihydroxy benzaldehyde (**1c**)] and ~1.0 mmol of 2ʹ,4ʹ-dihydroxyacetophenone (**2b**) (152.1 mg) were dissolved in 1.00 mL H_2_O, then 1.00 mL KOH 14 M was added. The flask containing the resulting mixture was suspended in the ultrasonic water bath at 80 °C for 8 h.

All above reactions were monitored by thin-layer chromatography (TLC) with the MeOH/CHCl_3_ (6–10 %). After completion, the reaction mixtures were quenched by acidification with HCl 3 M to pH ~5 and cooled to 0 °C to precipitate crude products, which were recrystallized with MeOH:H_2_O (1:3) to afford pure chalcones.

### General procedure for O-methylation (compound **1d**–**f**, **2d**–**f**, **8**, **9**, and **14**–**16**)

Dissolved ~1.0 mmol of the reactants [138.2 mg of 3,4-dihydroxybenzaldehyde (**1a**); 138.1 mg of 2,4-dihydroxybenzaldehyde (**1c**); 152.4 mg of 2ʹ,4ʹ-dihydroxyacetophenone (**2b**); 241.5 mg of 3,4-dihydroxychalcone (**3**); 272.1 mg of 3,4,2ʹ,4ʹ-tetrahydroxychalcone (**5**)] in 10.00 mL acetone, then added Na_2_CO_3_ (160.0 mg, 1.51 mmol). These were subsequently treated with CH_3_I in a fourfold amount corresponding to the moles of the hydroxyl group in the reactants (1.135 or 2.271 g). The mixture was stirred for 24–36 h at room temperature. Then the reaction mixture was acidified with HCl 1 M and extracted three times with ethyl acetate (20 mL × 3). Finally, flash column chromatography was used with EtOAc/*n*-hexane (20 %) to purify the products.

### General procedure for the synthesis of O-methylated chalcones (compounds **7**–**13**)

The benzaldehyde derivatives (**1a**, **1d**, **1e**, or **1f**) and the appropriate acetophenone derivatives (**2b**, **2d**, **2e**, or **2f**) were dissolved in 1.00 mL H_2_O (except for the experiment carried out with **1e** or **2f**, which was dissolved in 1.00 mL MeOH), then added 1.00 mL KOH 12 M. The flask containing the resulting mixture was suspended in ultrasonic water bath at 80 °C for 8 h. The desired products were obtained by the following work-up: the reaction mixtures were acidified with HCl 3 M to pH ~5; the solutions were allowed to cool slowly to 0 °C to precipitate crude products. These were recrystallized with MeOH:H_2_O (1:3) to afford pure chalcones.

#### 3ʹ-Caffeoyl-3,4,2ʹ-trihydroxy-4ʹ-methoxychalcone (**13**)

m.p. 200–201 °C. ^1^H-NMR (500 MHz, acetone-*d*_6_): 8.36 (d, *J* = 9.0, H–C(6ʹ)); 7.83 (*d*, *J* = 15.3, H–C(*β*)); 7.76 (*d*, *J* = 15.3, H–C(*α*)); 7.37 (*d*, *J* = 2.0, H–C(2)); 7.27 (*d*, *J* = 16.0, H–C(*β*ʹ)); 7.23 (*dd*, *J* = 9.0, 2.0, H–C(6)); 7.16 (*d*, *J* = 2.0, H–C(2′′)); 7.00 (*dd*, *J* = 9.0, 2.0, H–C(6′′)); 6.90 (*d*, *J* = 9.0, H–C(5)); 6.84 (*d*, *J* = 9.0, H–C(5′′)); 6.79 (*d*, *J* = 16.0, H–C(*α*ʹ)); 6.78 (*d*, *J* = 9.0, H–C(5ʹ)); 3.89 (*s*, MeO). ^13^C-NMR (125 MHz, acetone-*d*_6_): 193.3; 193.1; 164.3; 163.5; 150.4; 149.4; 146.8; 146.6; 146.5; 146.4; 133.4; 127.8; 127.7; 126.5; 123.9; 123.1; 118.9; 118.0; 116.5; 116.4; 116.3; 115.8; 115.3; 108.8; 56.6. HR-ESI–MS: *m/z* 447.1072 ([M–H]^−^, C_25_H_20_O_8_; 448.1158).

### General procedure for O-actylation (compound **17**)

Dissolved 50.0 mg of the compound **5** in 2.00 mL acetic anhydride, then added two drops of pyridine. The mixture was stirred for 1 h at room temperature. Finally, the crude product was precipitated by water addition, which was purified by using flash column chromatography with EtOAc/CHCl_3_ (0–20 %).

#### 3,4,4ʹ-Triacetoxy-2ʹ-hydroxychalcone (**17**)

m.p. 110–111 °C. ^1^H-NMR (500 MHz, acetone-*d*_6_): 8.35 (*d*, *J* = 9.0, H–C(6ʹ)); 8.06 (*d*, *J* = 15.5, H–C(*β*)); 7.94 (*d*, *J* = 15.5, H–C(*α*)); 7.83 (*dd*, *J* = 8.3, 2.0, H–C(6)); 7.80 (*d*, *J* = 2.1, H–C(2)); 7.38 (*d*, *J* = 8.3, H–C(5)); 6.79 (*dd*, *J* = 9.0, 2.1, H–C(5ʹ)); 6.77 (*d*, *J* = 2.1, H–C(3ʹ)); 2.31, 2.30, 2.29 (*s*, 3 AcO). ^13^C-NMR (125 MHz, acetone-*d*_6_): 194.1; 169.0; 168.7; 168.5; 158.2; 157.1; 145.6; 144.5; 144.0; 134.4; 132.9; 128.4; 125.1; 124.5; 122.6; 118.7; 114.0; 111.7; 20.6; 20.5; 12.1. HR-ESI–MS: *m/z* 397.0915 ([M–H]^−^, C_21_H_18_O_8_; 398.1002).

#### 4ʹ-Hydroxy-2ʹ-methoxyacetophenone (**2c**)

The reaction mixture consisting of 4.012 g polyphosphoric acid, 0.310 g of 3-methoxyphenol (2.5 mmol) and 0.21 mL of glacial acetic acid (3.78 mmol) was stirred at 60–70 °C for 30 min. The crude product was extracted three times with ethyl acetate (20 mL × 3). Used flash column chromatography with EtOAc/*n*-hexane (20 %) to purify the product **2c**, and the reaction yield was 30 %. Obtained **2c** together with two by-products **2d** and **2e**.

#### Assessment of xanthine oxidase inhibitory activity

Briefly, the XO inhibitory activity was assayed spectrophotometrically under aerobic conditions (Nguyen et al. 2005). The assay mixture consisting of 50 μL of test solution, 35 μL of 70 mM phosphate buffer (pH 7.5), and 30 μL of enzyme solution (0.01 units/mL in 70 mM phosphate buffer, pH 7.5) was prepared immediately before use. After preincubation at 25 °C for 15 min, the reaction was initiated by the addition of 60 μL of substrate solution (150 μM xanthine in the same buffer). The assay mixture was incubated at 25 °C for 30 min. The reaction was stopped by adding 25 μL of HCl 1 N, and the absorbance at 290 nm was measured with a *Shimadzu UV*-*1800*. A blank was prepared in the same way, but the enzyme solution was added to the assay mixture after adding HCl 1 N. One unit of xanthine oxidase is defined as the amount of enzyme required to produce 1 μmol of uric acid/min at 25 °C. XO inhibitory activity was expressed as the percentage inhibition of XO in the above assay system, calculated as (1 − *B/A*) × 100, where A and B are the activities of the enzyme without and with the test material. IC_50_ values were calculated from the mean values of data from four determinations. Allopurinol, a known inhibitor of XO, was used as a positive control.

## Conclusions

It is the first research on synthesis sappanchalcone (**7**) by Claisen–Schmidt condensation. This procedure was simple and generated fewer by-products than Heck coupling reaction followed by demethylation (Bianco et al. 2004). The overall yield of this procedure was 6.6 %, higher than that of reported procedure (4 %) (Bianco et al. 2004). Nine out of fifteen synthetic chalcones showed inhibitory activity (**3**; **5**–**8**; **10**–**13**). Compound **5**, **7**, **11** and **13** with IC_50_ values ranging from 2.4 to 4.3 μM displayed potent activity, comparing to allopurinol (IC_50_, 2.5 μM). This result suggests that these chalcone derivatives can be used as potential non-purine xanthine oxidase inhibitors. Structure–activity relationship was also proposed.

## Additional file

10.1186/s40064-016-3485-6 The NMR data of all synthetic compounds and HRMS spectra of two novel compound **13** and **17**.
