# Supplementary material for: Design and synthesis of chalcone derivatives as potential non-purine xanthine oxidase inhibitors
Source: Springerplus. 2016 Oct 13;5(1):1789. doi: 10.1186/s40064-016-3485-6 (PMC5063830; doi:10.1186/s40064-016-3485-6)
Supplement: Supplementary file 1 — 10.1186/s40064-016-3485-6 The NMR data of all synthetic compounds and HRMS spectra of two novel compound 13 and 17. [file 40064_2016_3485_MOESM1_ESM.pdf]

Supplementary Files for

Design and Synthesis of Chalcone Derivatives as Potential Non-Purine Xanthine Oxidase Inhibitors

Trung Huu Bui, Nhan Trung Nguyen, Phu Hoang Dang, Hai Xuan Nguyen, Mai Thanh Thi Nguyen

Figure S1. *^{1}H-NMR spectrum of the compound* ***1d*** *(500 MHz – CD_3COCD_3)*

Figure S2. *^{1}H-NMR spectrum of the compound* ***1e*** *(500 MHz – CD_3COCD_3)*

Figure S3. *^{1}H-NMR spectrum of the compound* ***1f*** *(500 MHz – $CDCl_3$)*

Figure S4.1. *^{1}H-NMR spectrum of the compound* ***2c*** *(500 MHz – $CDCl_3$)*

Figure S4.2. *^{13}C-NMR spectrum of the compound* ***2c*** *(125 MHz – $CDCl_3$)*

Figure S5.1. *^{1}H-NMR spectrum of the compound* ***2d*** *(500 MHz – CD_3COCD_3)*

Figure S5.2. *^{13}C-NMR spectrum of the compound* ***2d*** *(125 MHz – CD_3COCD_3)*

Figure S6.1. *^{1}H-NMR spectrum of the compound* ***2e*** *(500 MHz – $CDCl_3$)*

Figure S6.2. *^{13}C-NMR spectrum of the compound* ***2e*** *(125 MHz – $CDCl_3$)*

Figure S7. *^{1}H-NMR spectrum of the compound* ***2f*** *(500 MHz – CD_3COCD_3)*

Figure S8.1. *^{1}H-NMR spectrum of the compound* ***3*** *(500 MHz – CD_3OD)*

Figure S8.2. *^{13}C-NMR spectrum of the compound* ***3*** *(125 MHz – CD_3OD)*

Figure S9.1. *^{1}H-NMR spectrum of the compound* ***4*** *(500 MHz – CD_3OD)*

Figure S9.2. *^{13}C-NMR spectrum of the compound* ***4*** *(125 MHz – CD_3OD)*

Figure S10.1. *^{1}H-NMR spectrum of the compound* ***5*** *(500 MHz – CD_3OD)*

Figure S10.2. *^{13}C-NMR spectrum of the compound* ***5*** *(125 MHz – CD_3OD)*

Figure S11.1. *^{1}H-NMR spectrum of the compound* ***6*** *(500 MHz – CD_3OD)*

Figure S11.2. *^{13}C-NMR spectrum of the compound* ***6*** *(125 MHz – CD_3OD)*

Figure S12.1. *^{1}H-NMR spectrum of the compound* ***7*** *(500 MHz – CD_3COCD_3)*

Figure S12.2. *^{13}C-NMR spectrum of the compound* ***7*** *(125 MHz – CD_3COCD_3)*

Figure S13.1. *^{1}H-NMR spectrum of the compound* ***8*** *(500 MHz – CD_3COCD_3)*

Figure S13.2. *^{13}C-NMR spectrum of the compound* ***8*** *(125 MHz – CD_3COCD_3)*

Figure S14.1. *^{1}H-NMR spectrum of the compound* ***9*** *(500 MHz – CD_3COCD_3)*

Figure S14.2. *^{13}C-NMR spectrum of the compound* ***9*** *(125 MHz – CD_3COCD_3)*

Figure S15.1. *^{1}H-NMR spectrum of the compound* ***10*** *(500 MHz – CD_3COCD_3)*

Figure S15.2. *^{13}C-NMR spectrum of the compound* ***10*** *(125 MHz – CD_3COCD_3)*

Figure S16.1. *^{1}H-NMR spectrum of the compound* ***11*** *(500 MHz – CD_3COCD_3)*

Figure S16.2. *^{13}C-NMR spectrum of the compound* ***11*** *(125 MHz – CD_3COCD_3)*

Figure S17.1. *^{1}H-NMR spectrum of the compound* ***12*** *(500 MHz – CD_3COCD_3)*

Figure S17.2. *^{13}C-NMR spectrum of the compound* ***12*** *(125 MHz – CD_3COCD_3)*

Figure S18.1. *^{1}H-NMR spectrum of the compound* ***13*** *(500 MHz – CD_3COCD_3)*

Figure S18.2. *^{13}C-NMR spectrum of the compound* ***13*** *(125 MHz – CD_3COCD_3)*

Figure S18.3. *HSQC-NMR spectrum of the compound* ***13***

Figure S18.4. *HMBC-NMR spectrum of the compound* ***13***

Figure S18.5. *HR-ESI-MS spectrum of the compound* ***13***

Figure S19.1. *^{1}H-NMR spectrum of the compound* ***14*** *(500 MHz – CD_3OD)*

Figure S19.2. *^{13}C-NMR spectrum of the compound* ***14*** *(125 MHz – CD_3OD)*

Figure S20.1. *^{1}H-NMR spectrum of the compound* ***15*** *(500 MHz – CD_3OD)*

Figure S20.2. *^{13}C-NMR spectrum of the compound* ***15*** *(125 MHz – CD_3OD)*

Figure S21.1. *^{1}H-NMR spectrum of the compound* ***16*** *(500 MHz – CD_3COCD_3)*

Figure S21.2. *^{13}C-NMR spectrum of the compound* ***16*** *(125 MHz – CD_3COCD_3)*

Figure S22.1. *^{1}H-NMR spectrum of the compound* ***17*** *(500 MHz – CD_3COCD_3)*

Figure S22.2. *^{13}C-NMR spectrum of the compound* ***17*** *(125 MHz – CD_3COCD_3)*

Figure S22.3. *HR-ESI-MS spectrum of the compound* ***17***

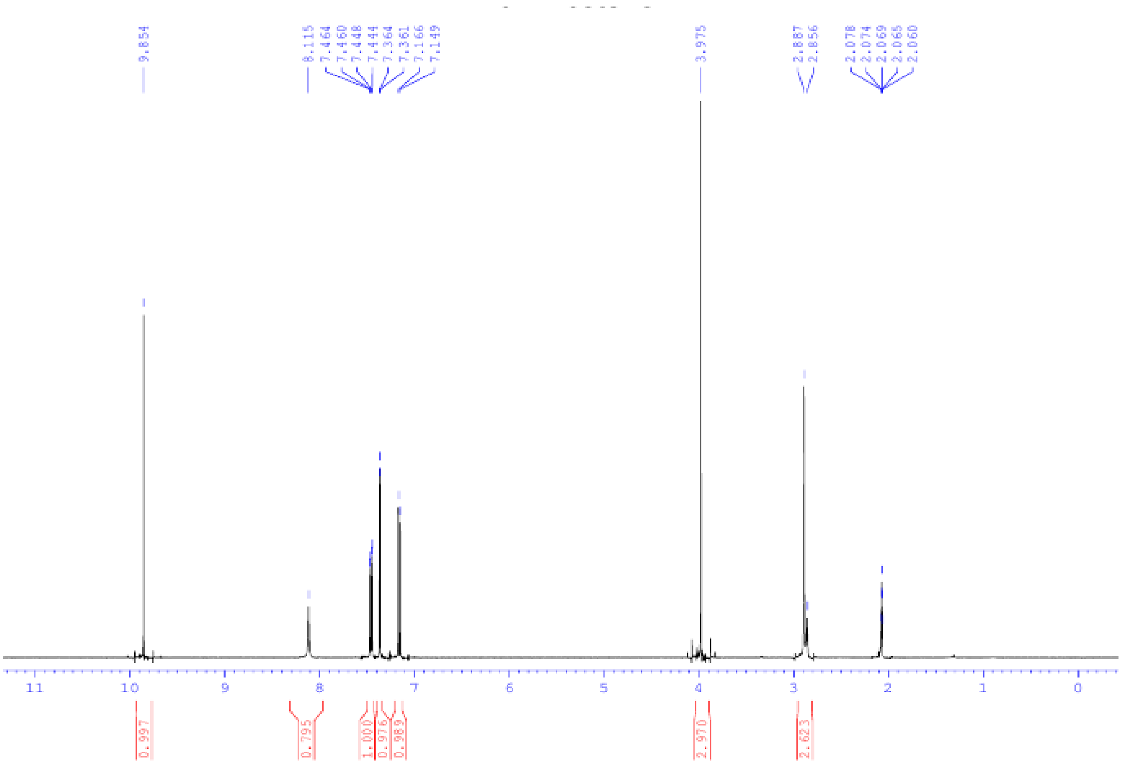

Figure S1. ^{1}H-NMR spectrum of the compound **1d** (500 MHz – CD_3COCD_3)

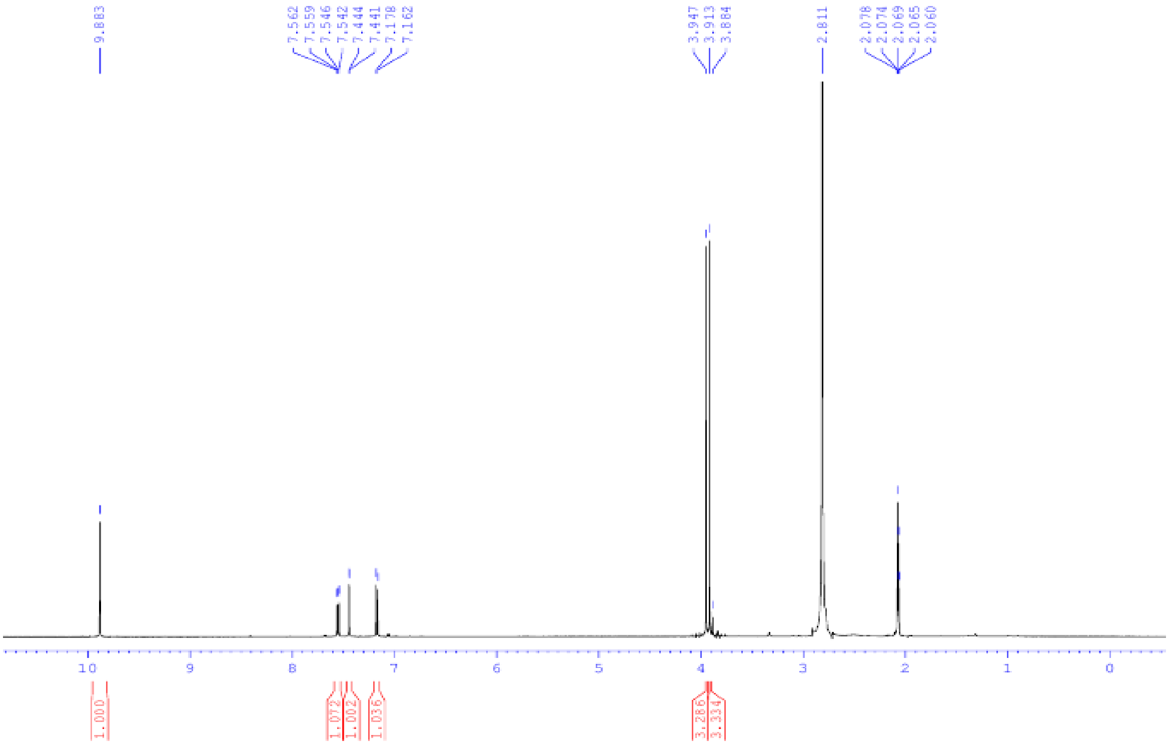

Figure S2. ^{1}H-NMR spectrum of the compound **1e** (500 MHz – CD_3COCD_3)

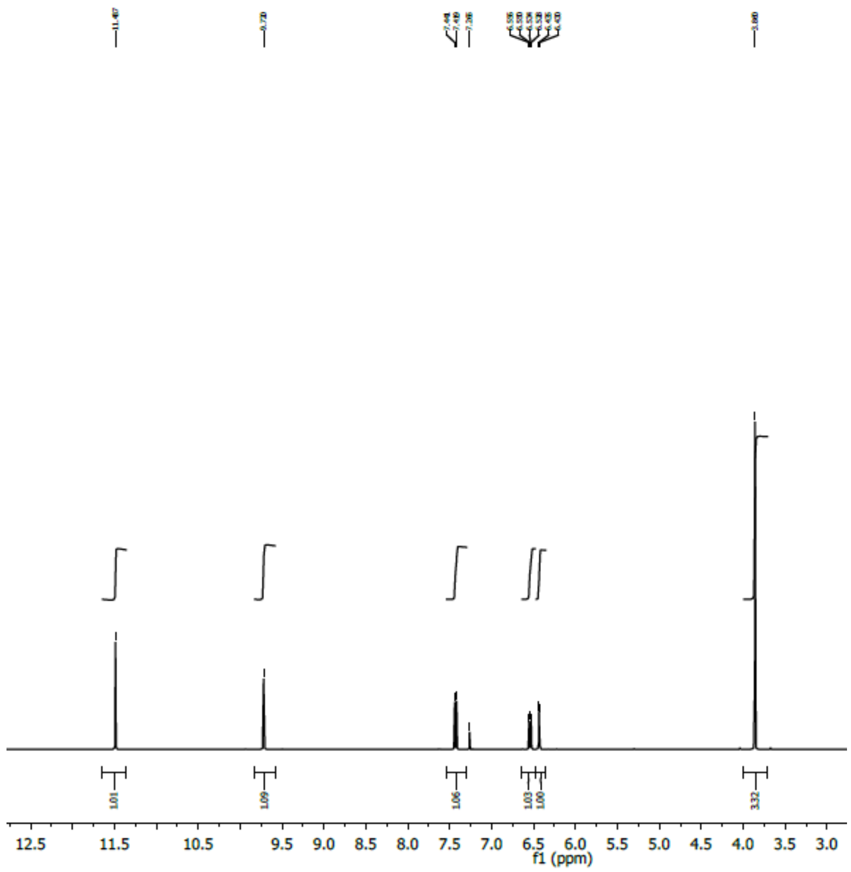

Figure S3. ^{1}H-NMR spectrum of the compound **1f** (500 MHz – $CDCl_3$)

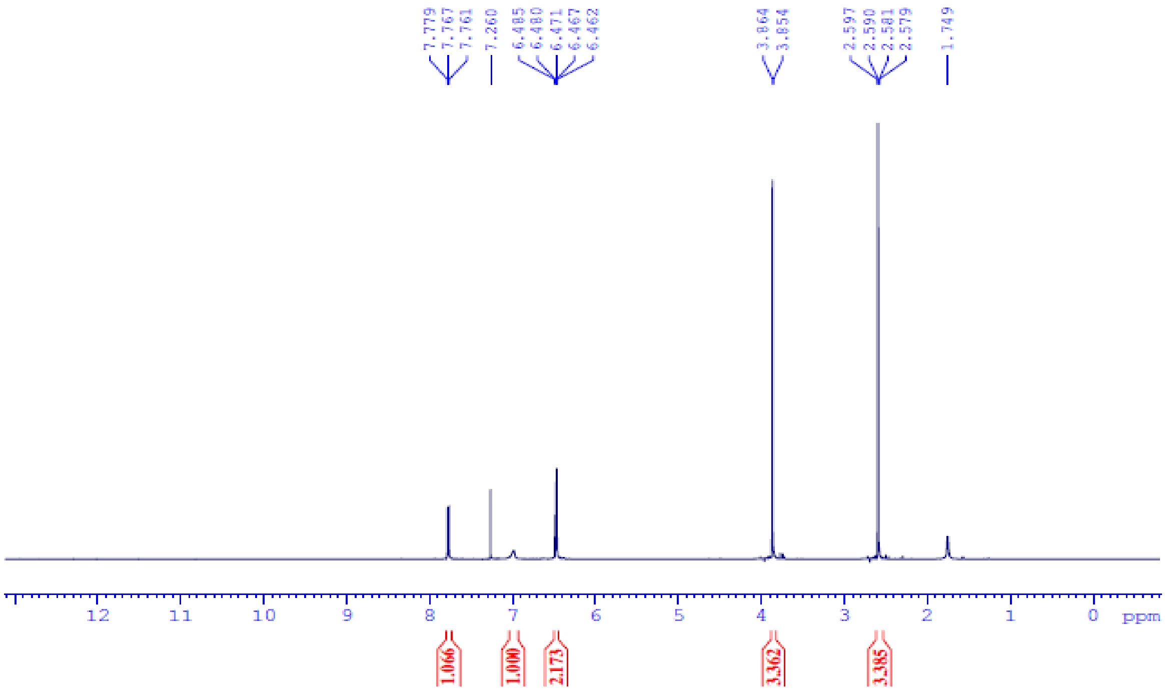

Figure S4.1. ^{1}H-NMR spectrum of the compound **2c** (500 MHz – $CDCl_3$)

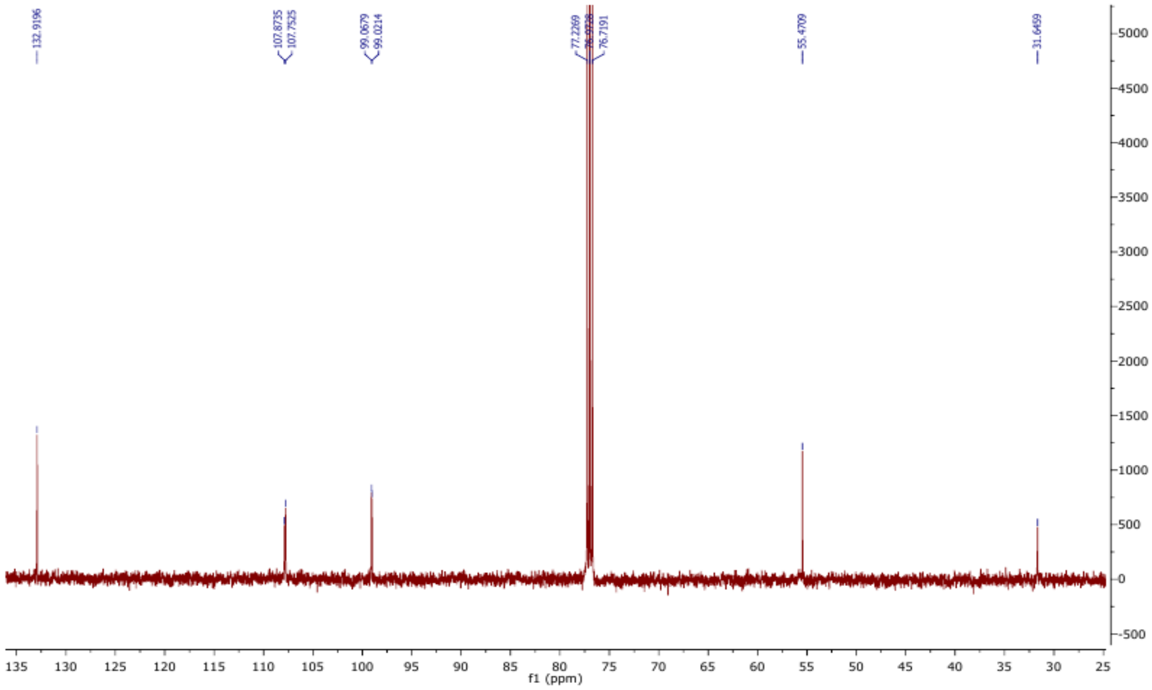

Figure S4.2. ^{13}C-NMR spectrum of the compound **2c** (125 MHz – $CDCl_3$)

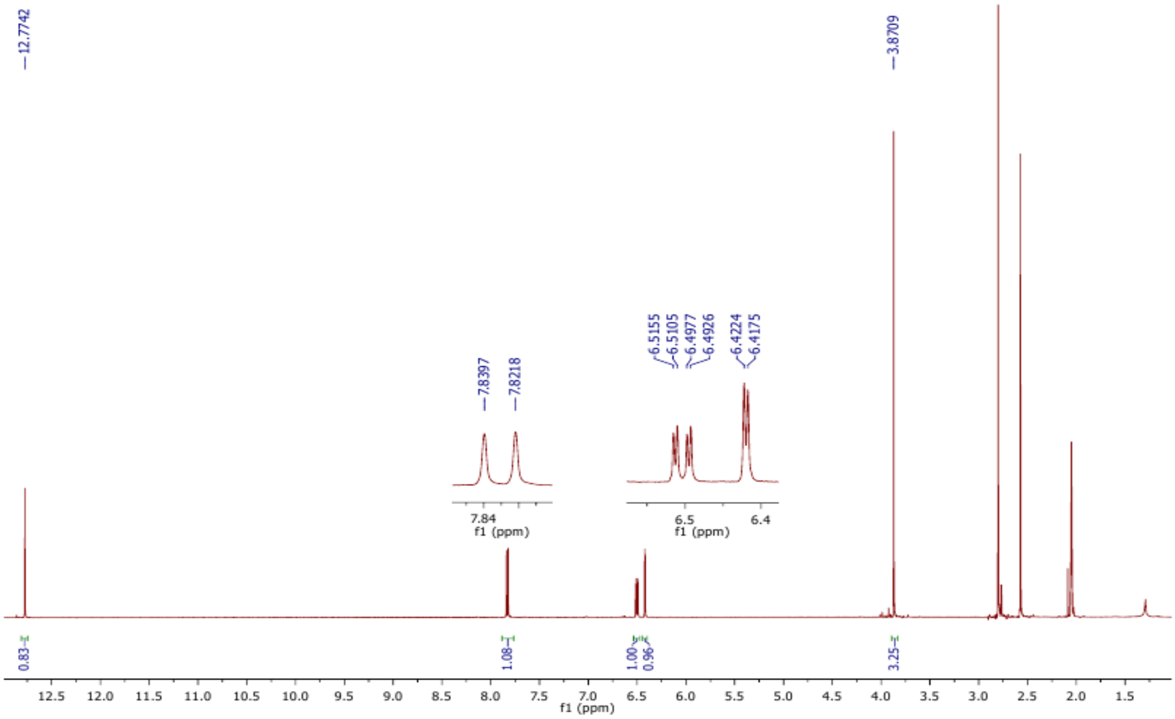

Figure S5.1. ^{1}H-NMR spectrum of the compound **2d** (500 MHz – CD_3COCD_3)

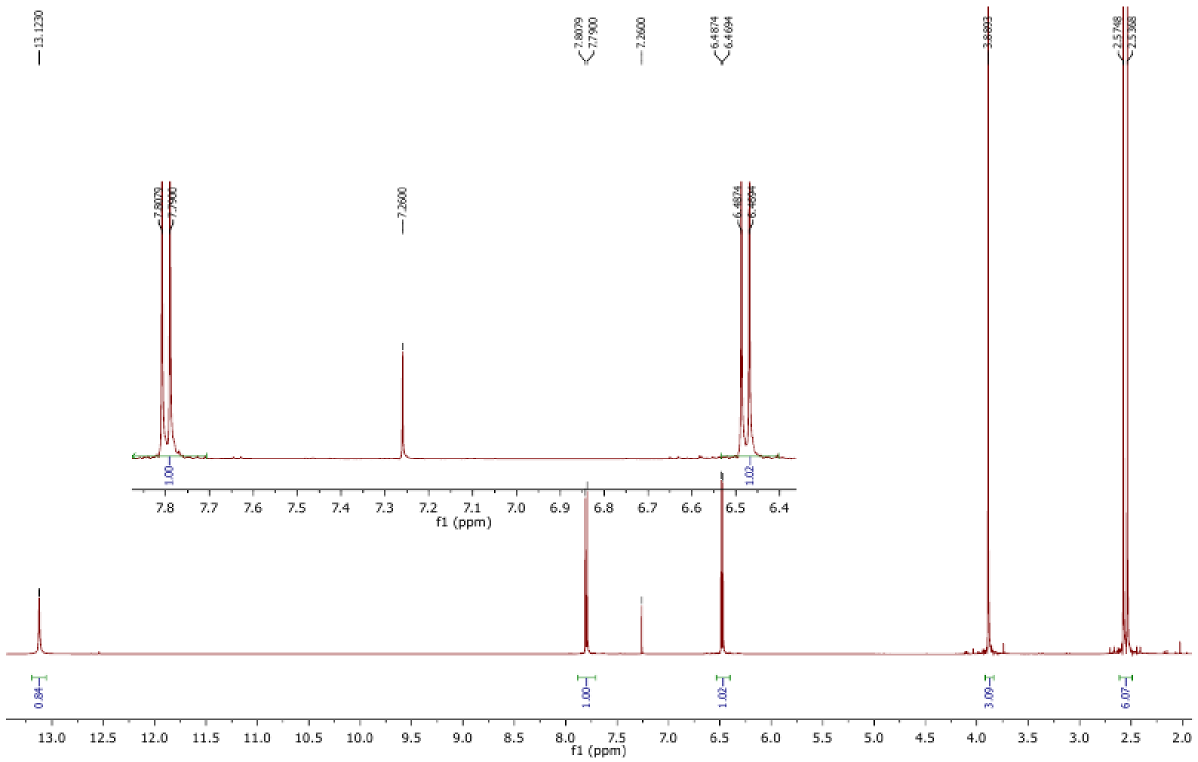

Figure S5.2. ^{13}C-NMR spectrum of the compound **2d** (125 MHz – CD_3COCD_3)

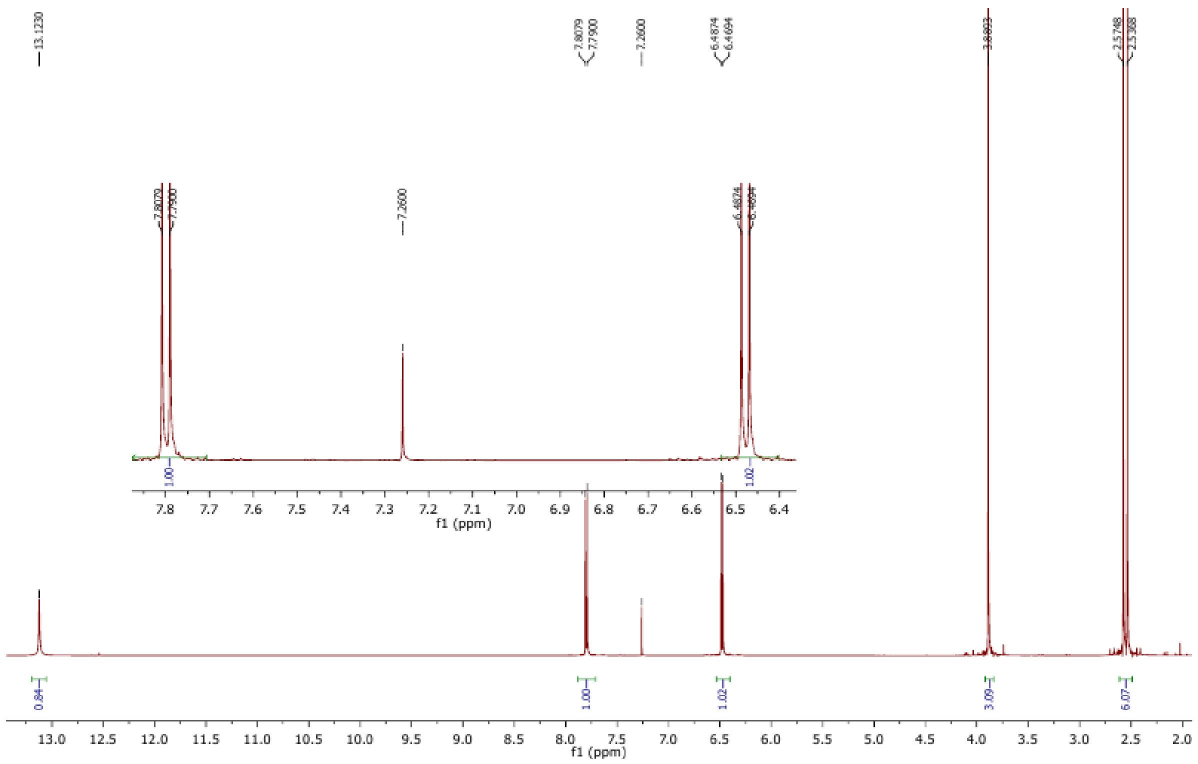

Figure S6.1. ^{1}H-NMR spectrum of the compound **2e** (500 MHz – $CDCl_3$)

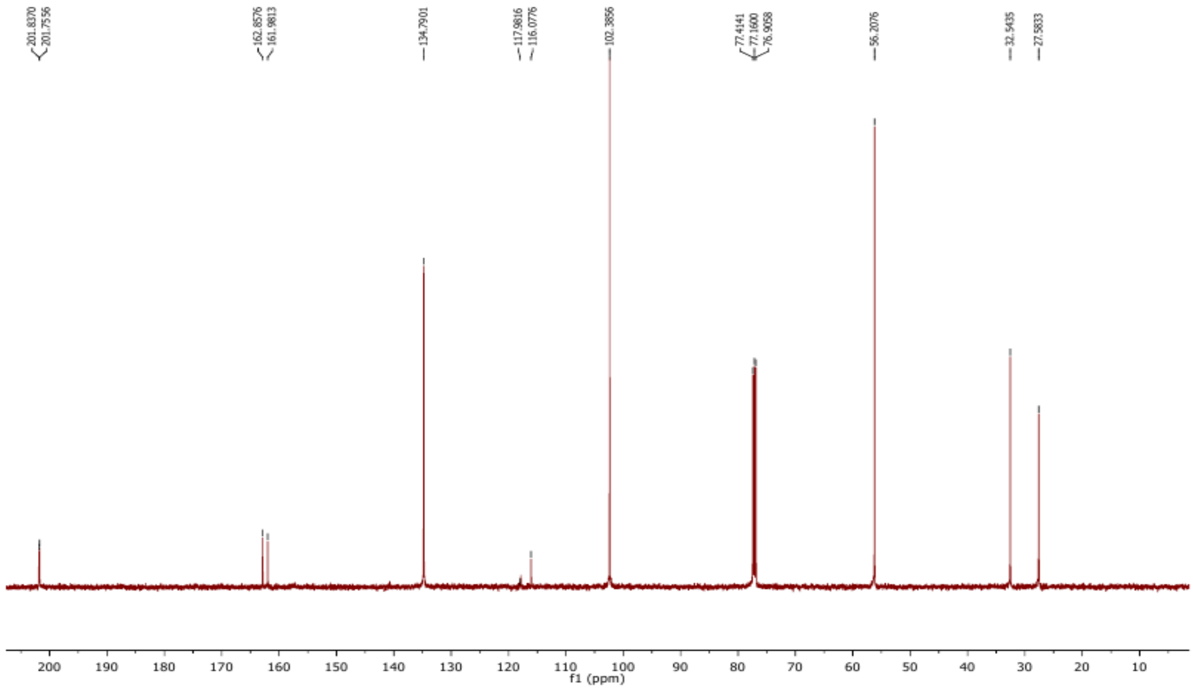

Figure S6.2. ^{13}C-NMR spectrum of the compound **2e** (125 MHz – $CDCl_3$)

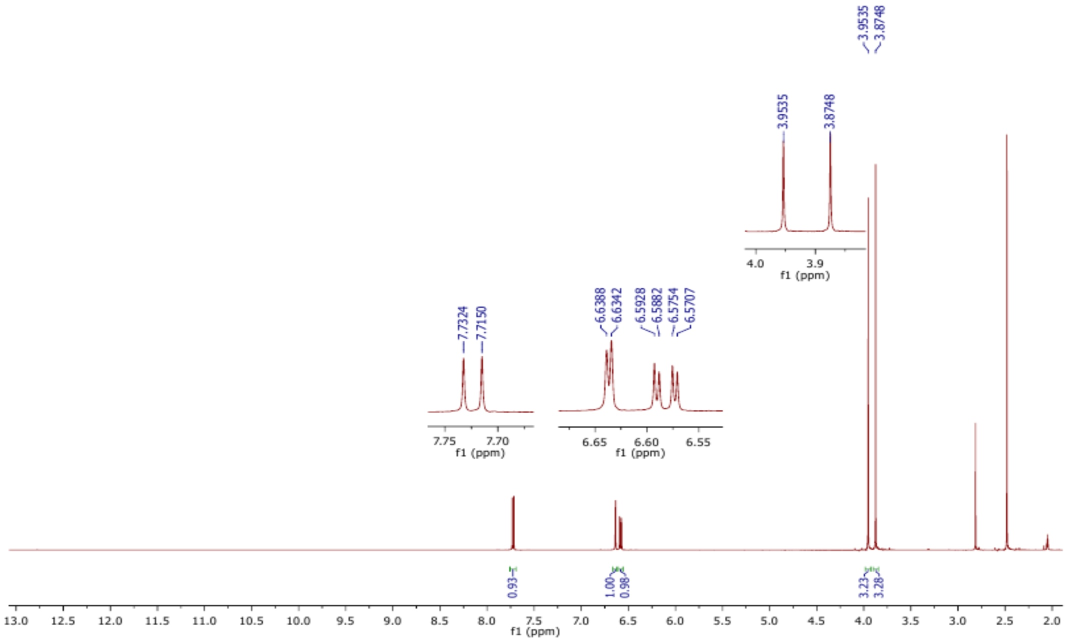

Figure S7. ^{1}H-NMR spectrum of the compound **2f** (500 MHz – CD_3COCD_3)

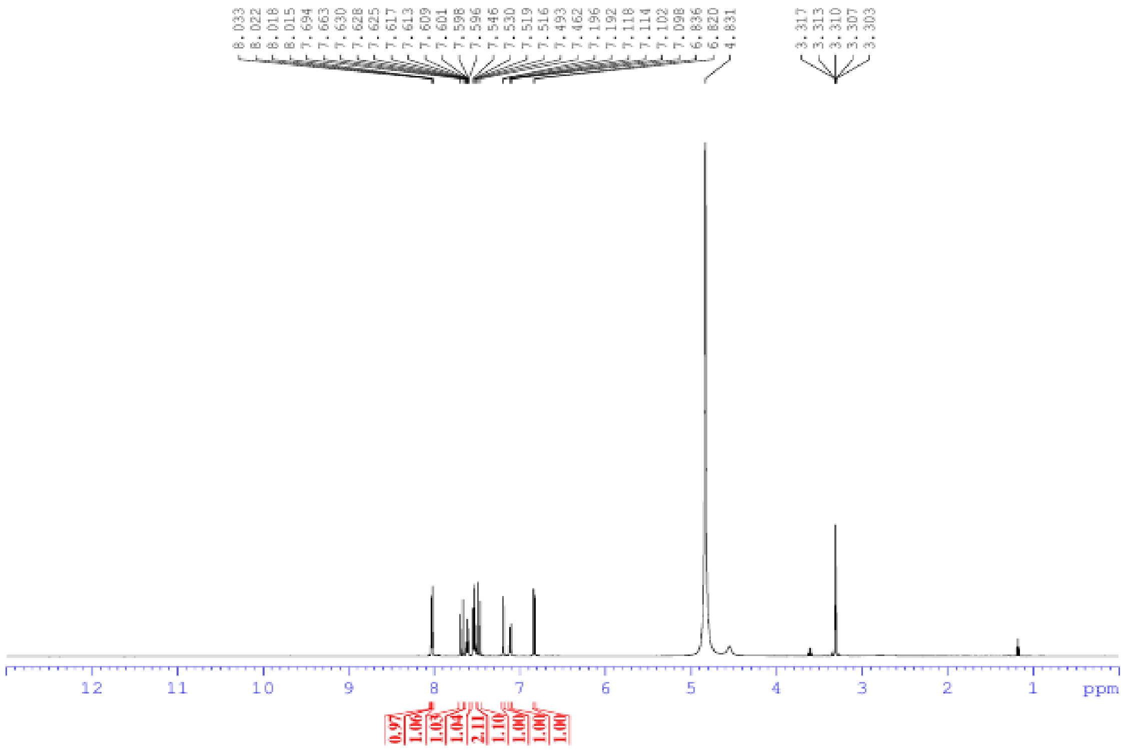

Figure S8.1. ^{1}H-NMR spectrum of the compound **3** (500 MHz – CD_3OD)

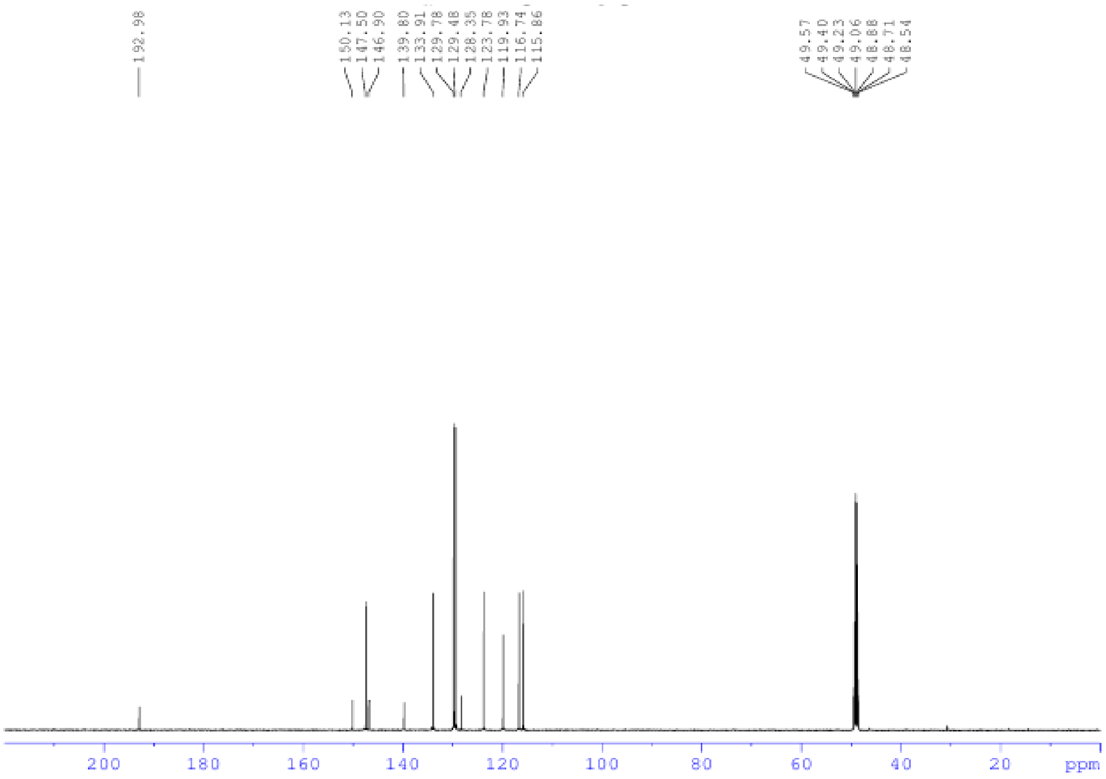

Figure S8.2. ^{13}C-NMR spectrum of the compound **3** (125 MHz – CD_3OD)

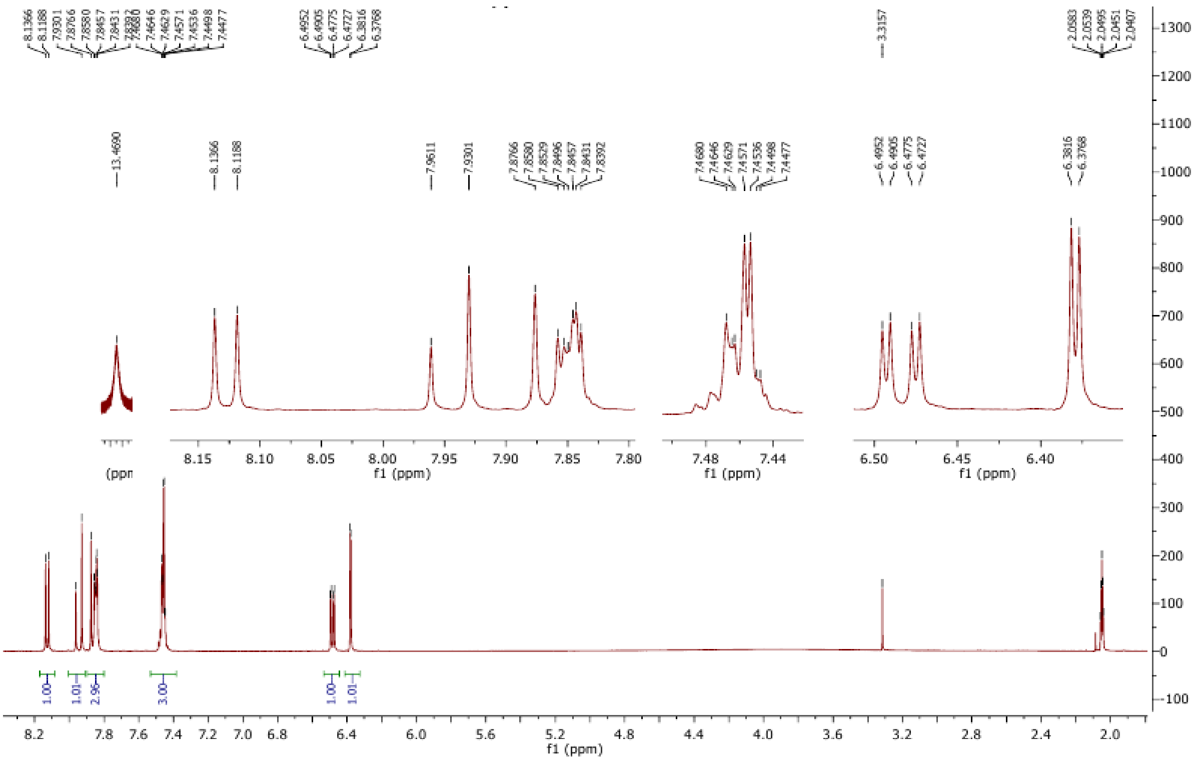

Figure S9.1. ^{1}H-NMR spectrum of the compound **4** (500 MHz – CD_3OD)

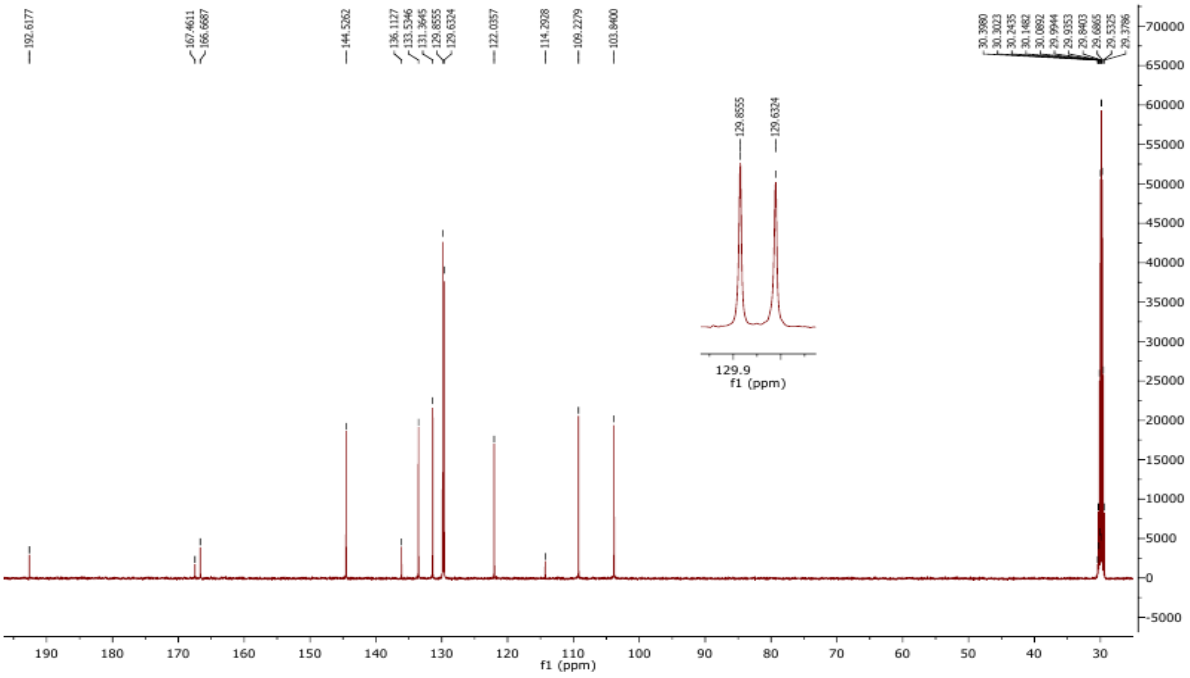

Figure S9.2. ^{13}C-NMR spectrum of the compound **4** (125 MHz – CD_3OD)

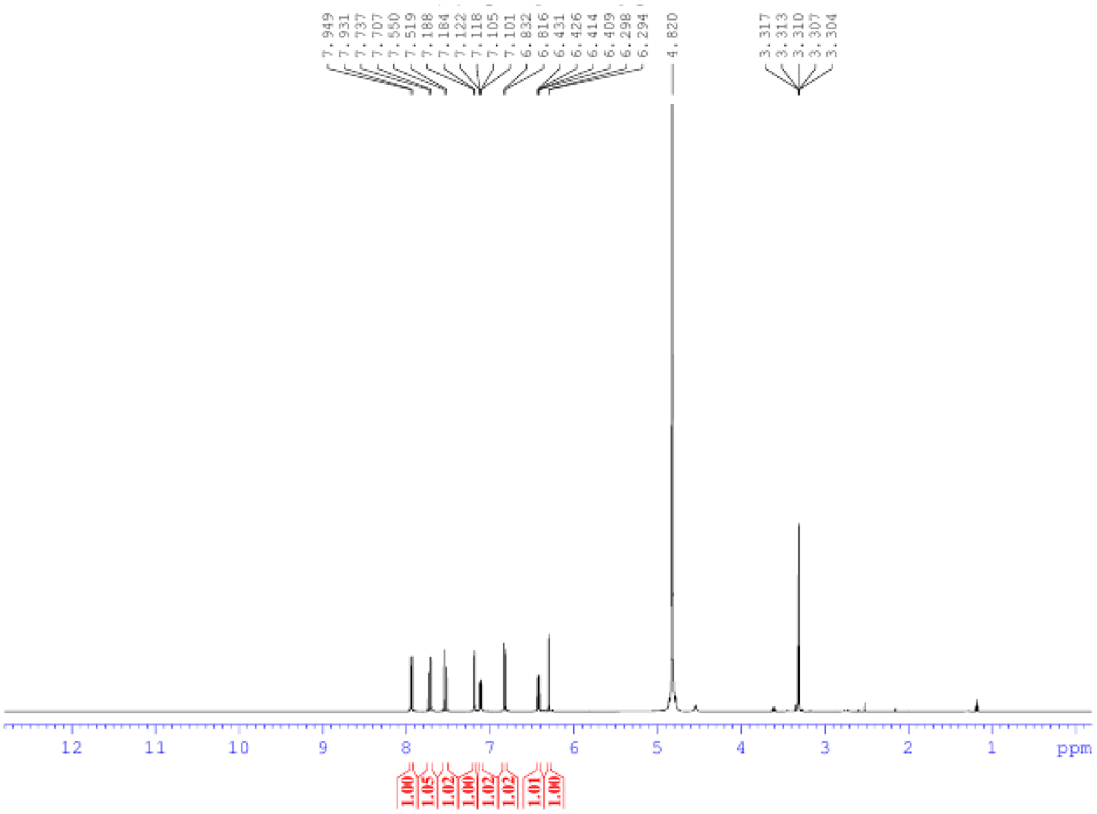

Figure S10.1. ^{1}H-NMR spectrum of the compound **5** (500 MHz – CD_3OD)

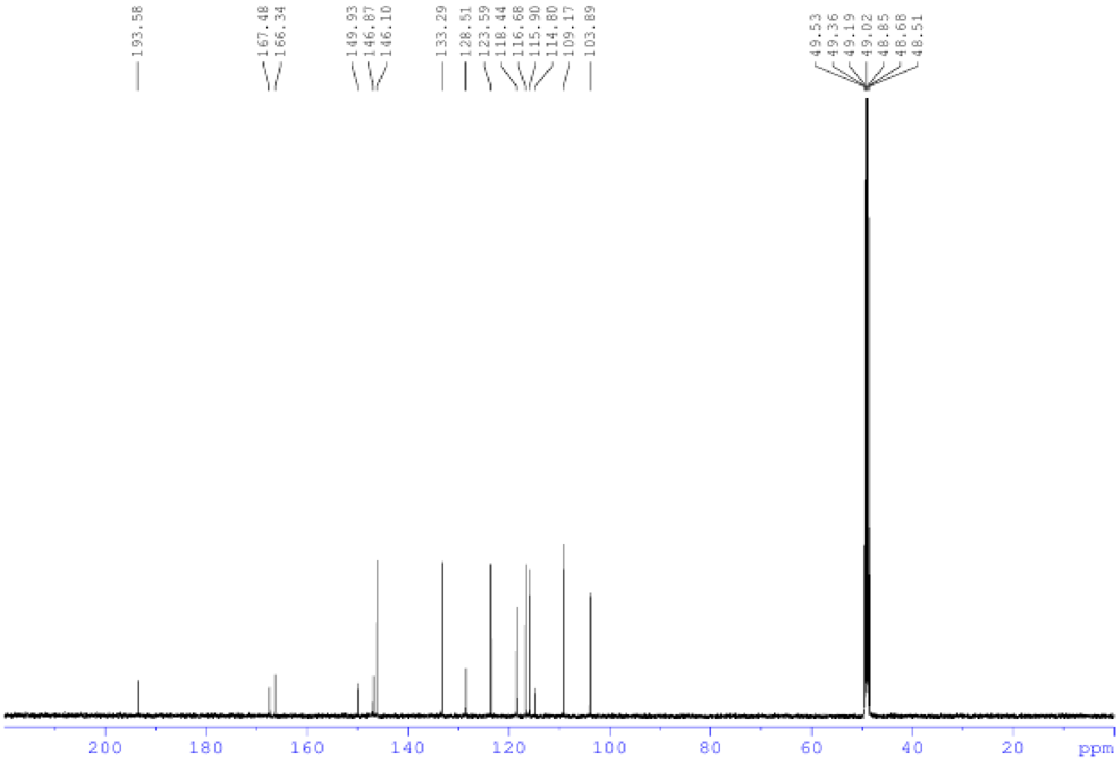

Figure S10.2. ^{13}C-NMR spectrum of the compound **5** (125 MHz – CD_3OD)

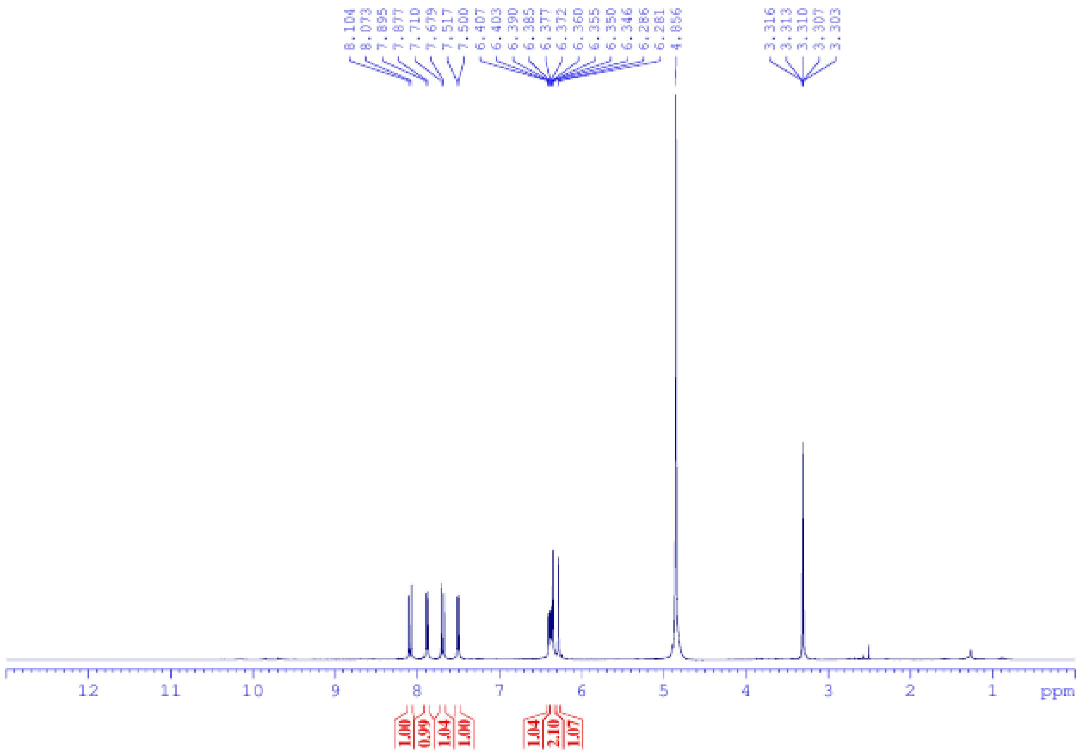

Figure S11.1. ^{1}H-NMR spectrum of the compound **6** (500 MHz – CD_3OD)

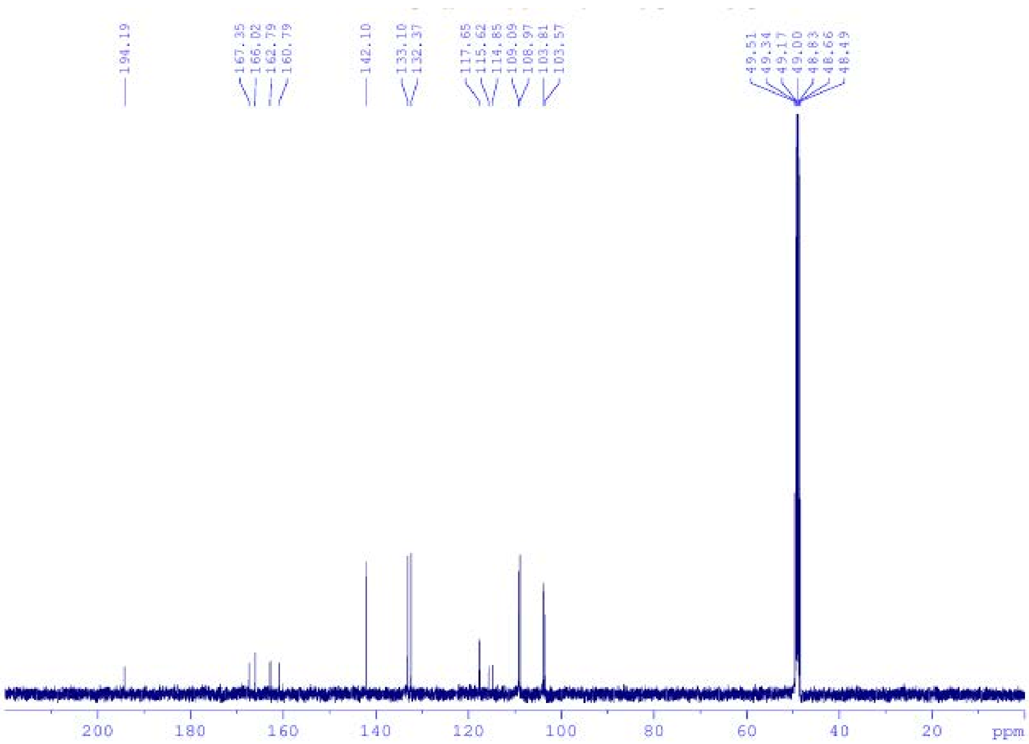

Figure S11.2. ^{13}C-NMR spectrum of the compound **6** (125 MHz – CD_3OD)

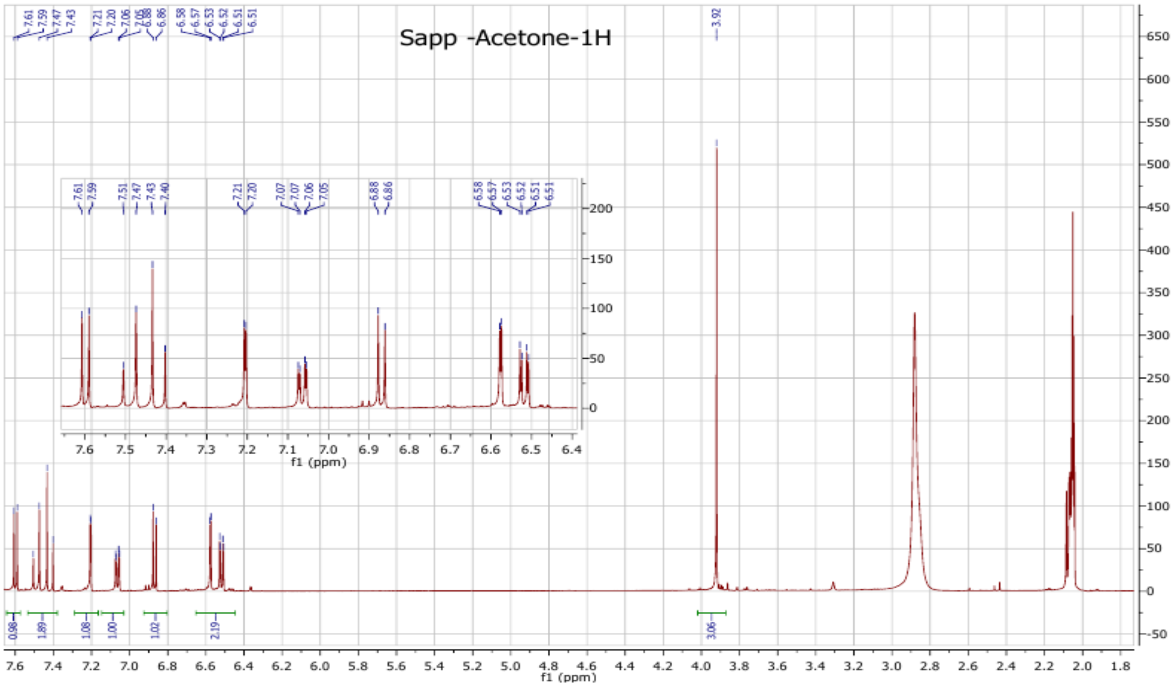

Figure S12.1. ^{1}H-NMR spectrum of the compound **7** (500 MHz – CD_3COCD_3)

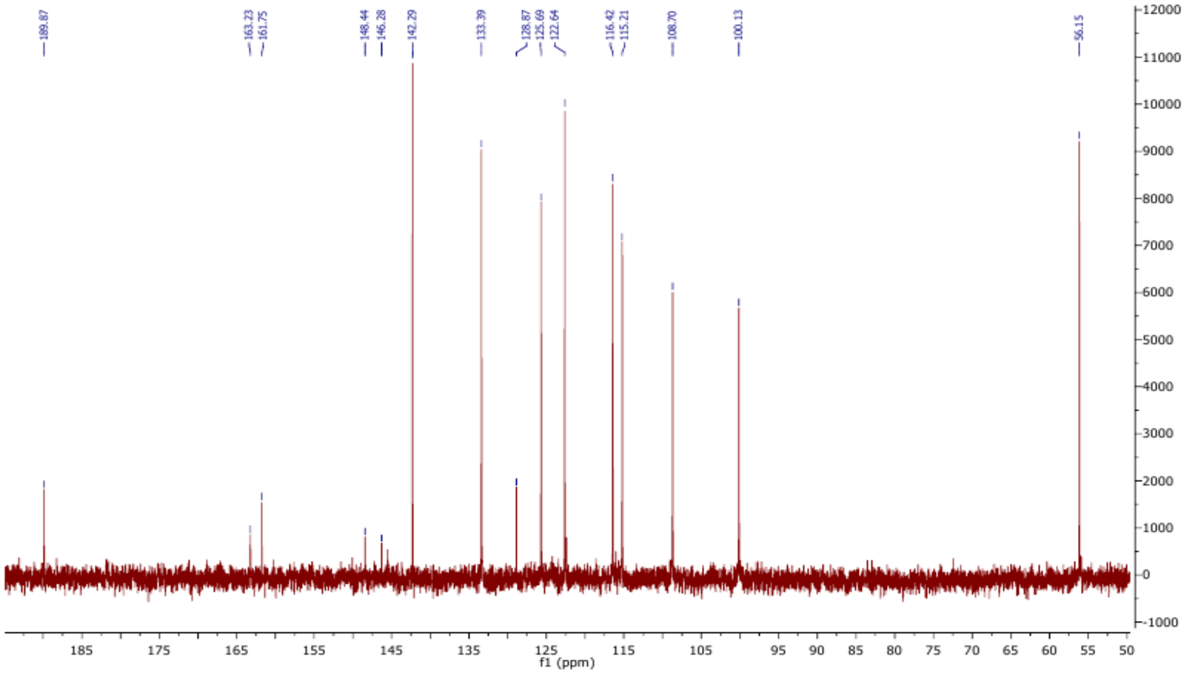

Figure S12.2. ^{13}C-NMR spectrum of the compound **7** (125 MHz – CD_3COCD_3)

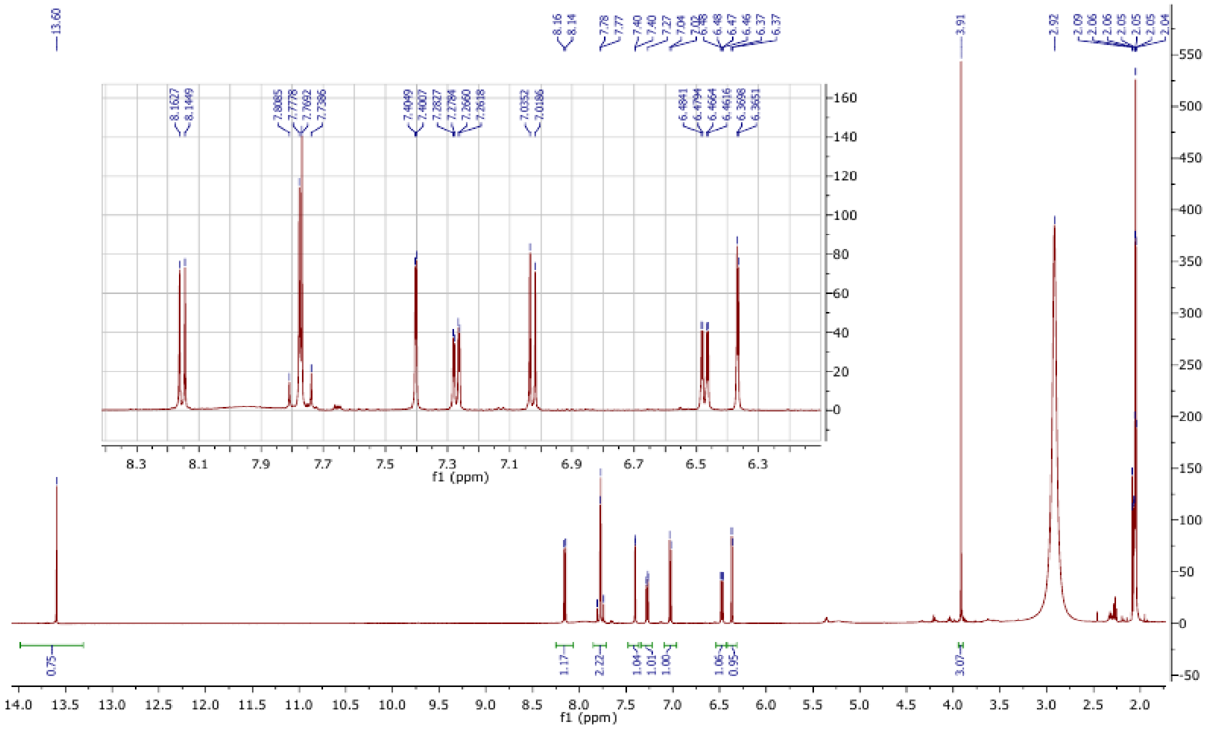

Figure S13.1. ^{1}H-NMR spectrum of the compound **8** (500 MHz – CD_3COCD_3)

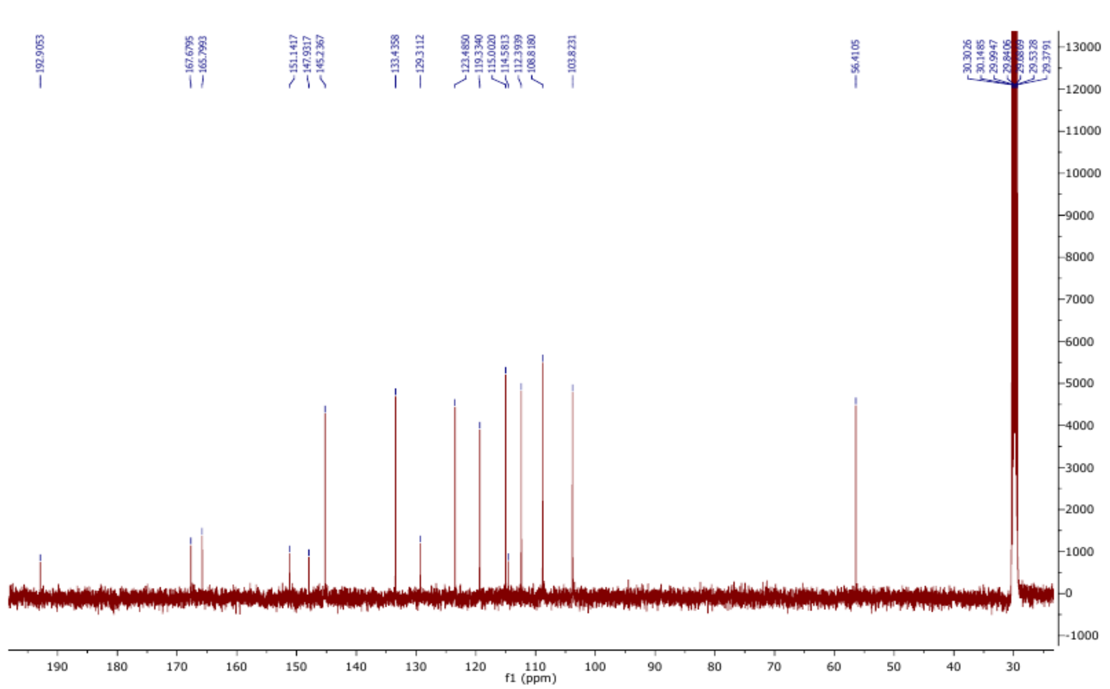

Figure S13.2. ^{13}C-NMR spectrum of the compound **8** (125 MHz – CD_3COCD_3)

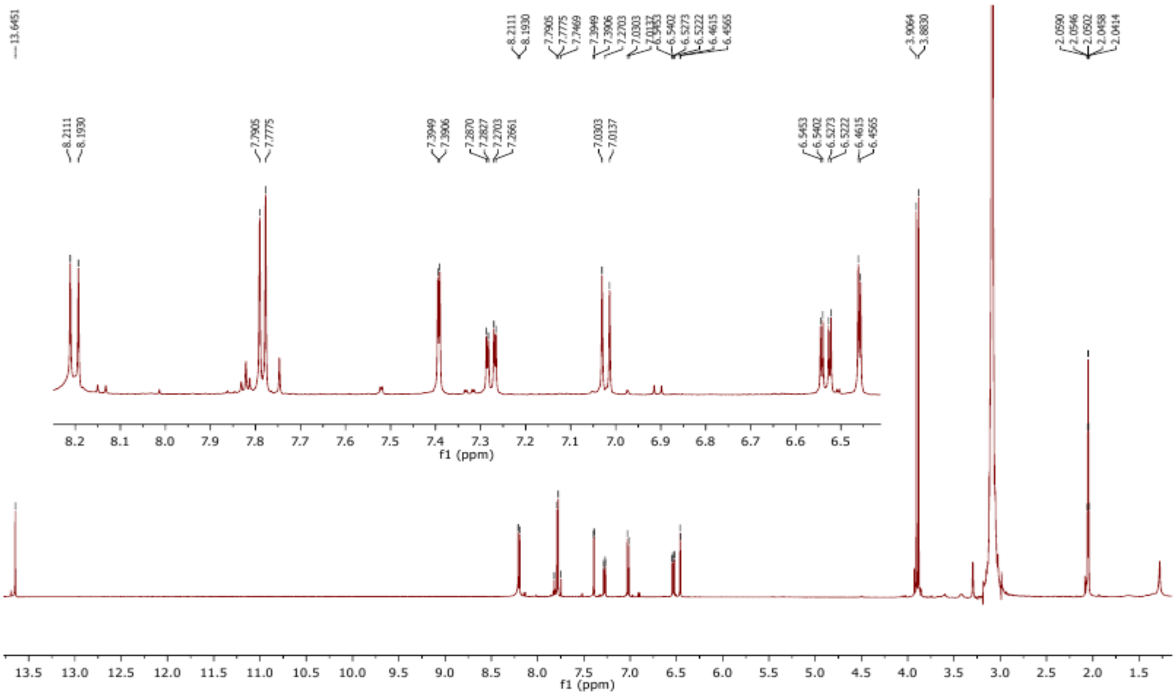

Figure S14.1. ^{1}H-NMR spectrum of the compound **9** (500 MHz – CD_3COCD_3)

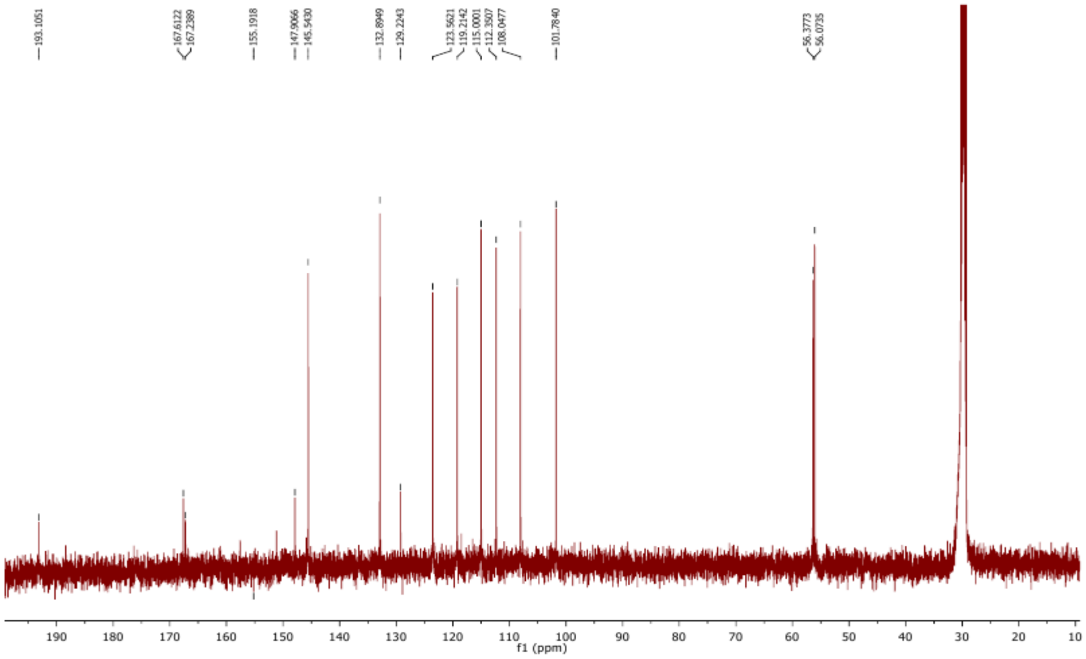

Figure S14.2. ^{13}C-NMR spectrum of the compound **9** (125 MHz – CD_3COCD_3)

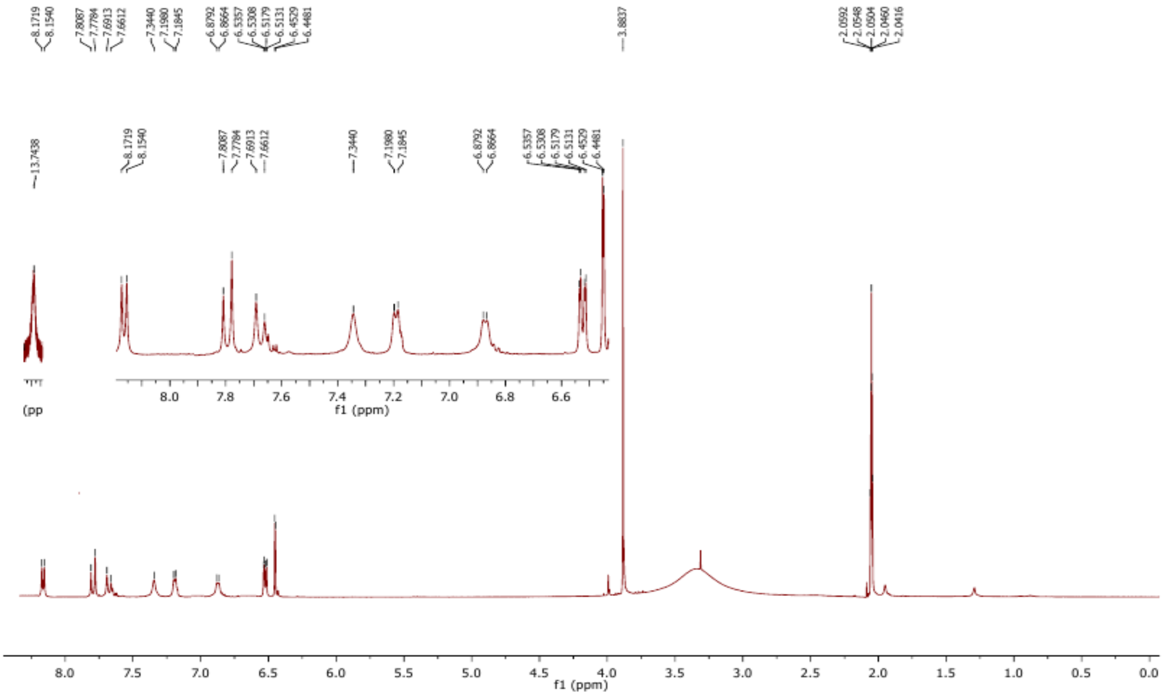

Figure S15.1. ^{1}H-NMR spectrum of the compound **10** (500 MHz – CD_3COCD_3)

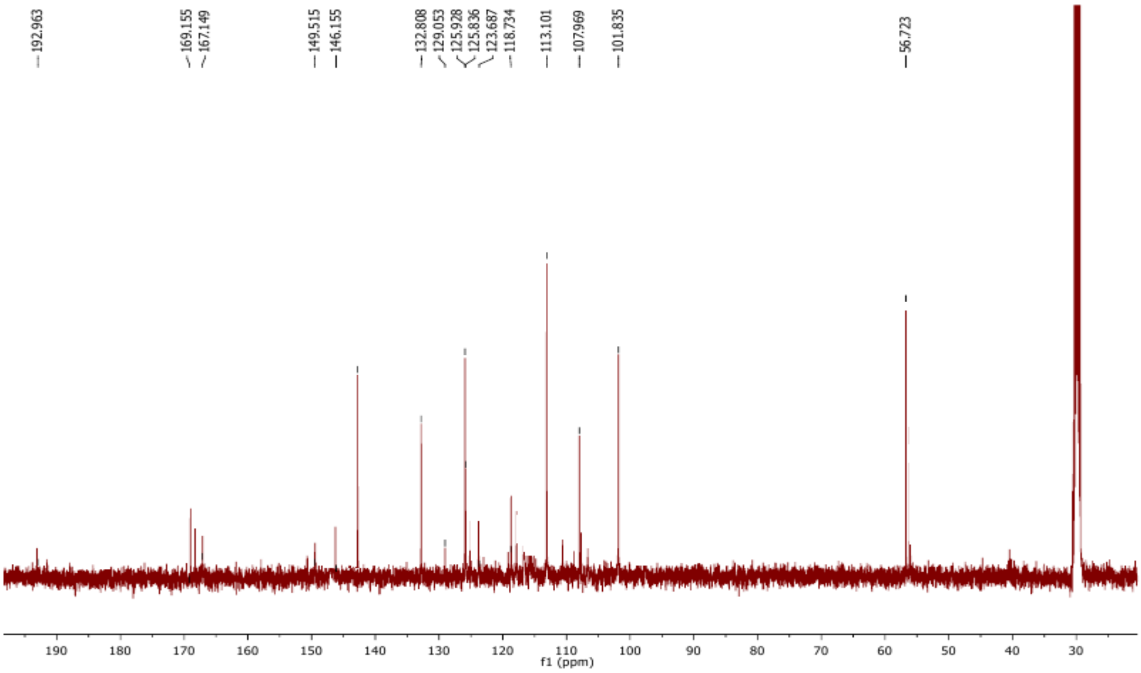

Figure S15.2. ^{13}C-NMR spectrum of the compound **10** (125 MHz – CD_3COCD_3)

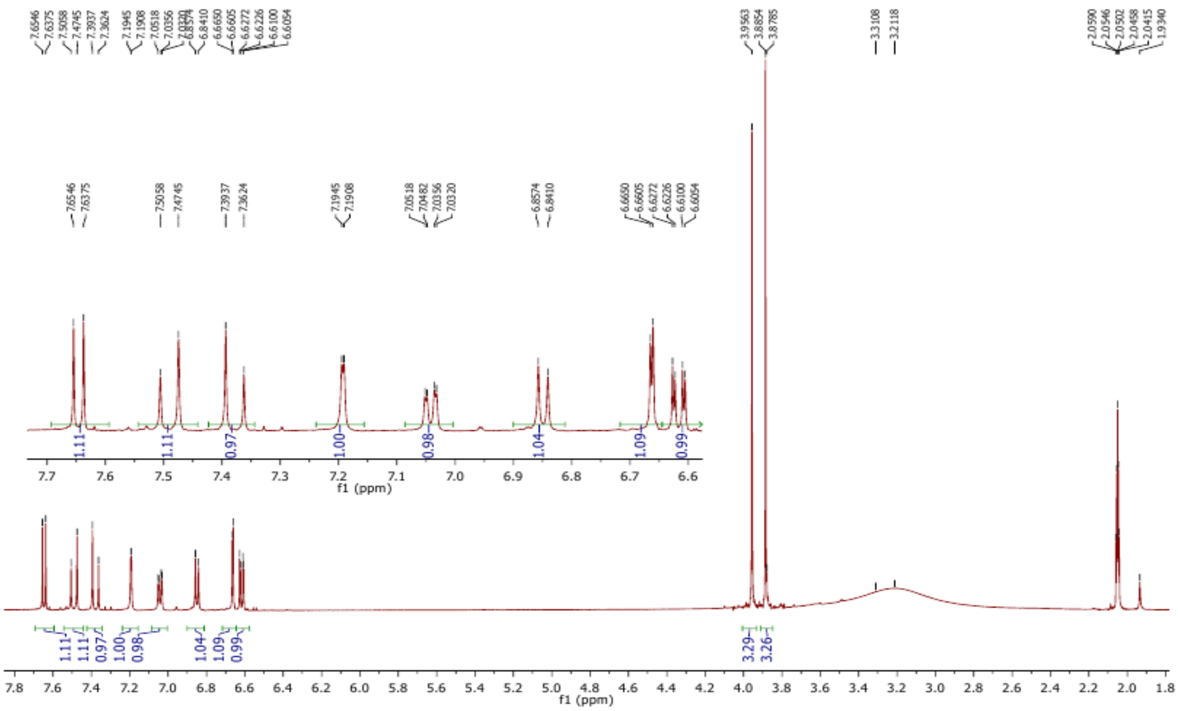

Figure S16.1. ^{1}H-NMR spectrum of the compound **11** (500 MHz – CD_3COCD_3)

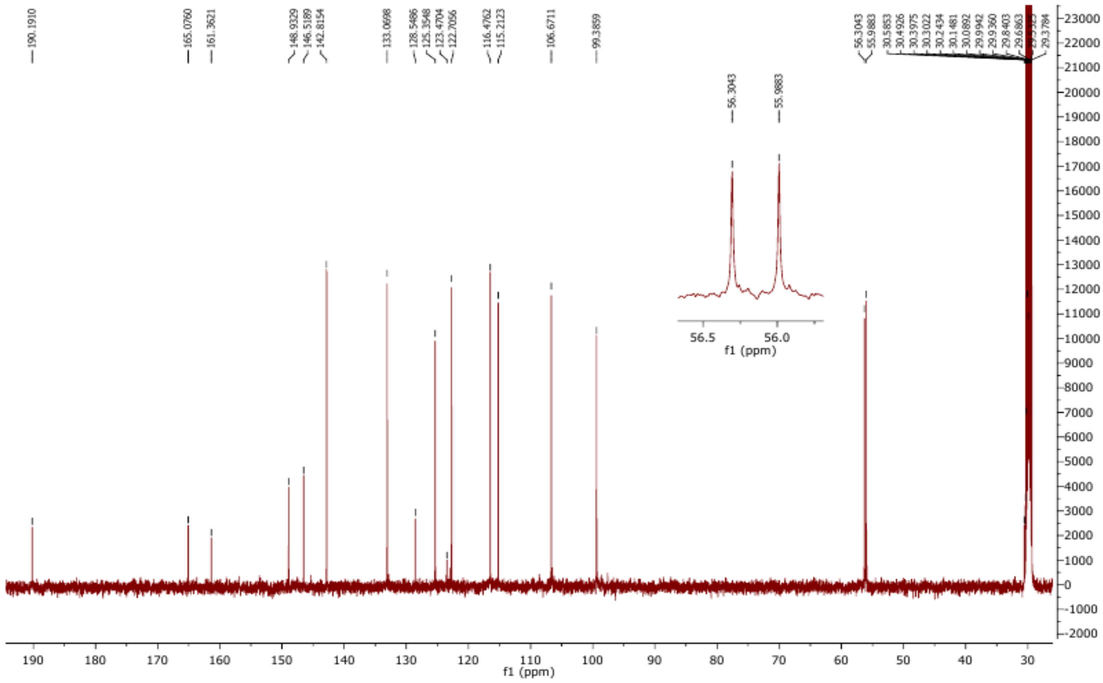

Figure S16.2. ^{13}C-NMR spectrum of the compound **11** (125 MHz – CD_3COCD_3)

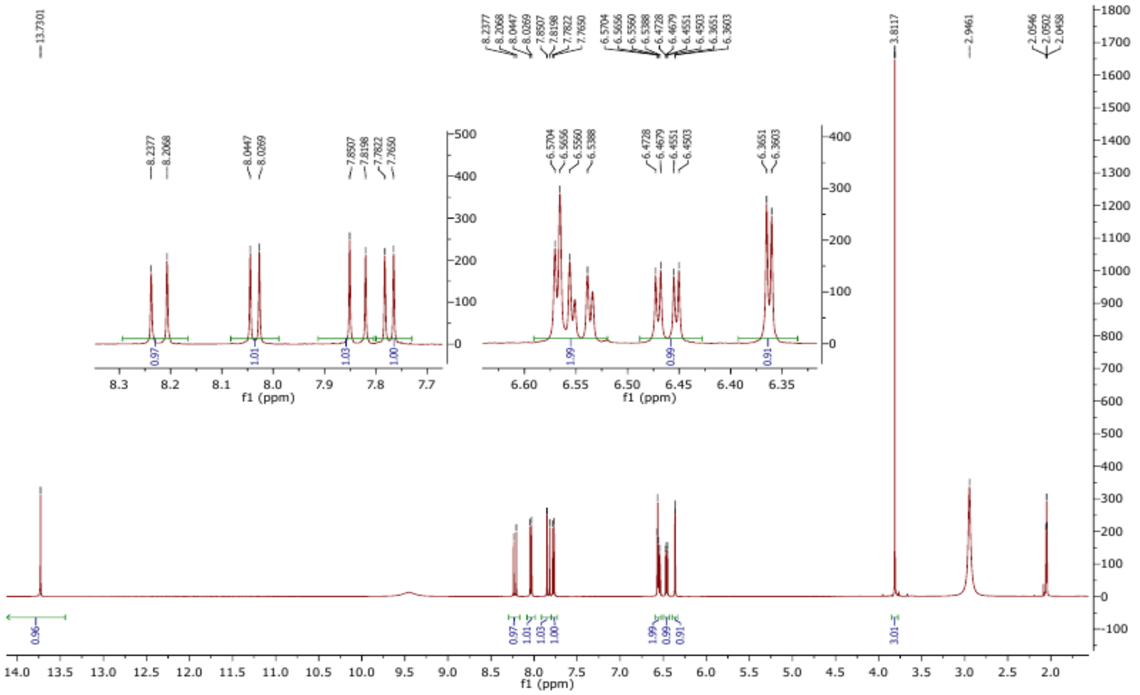

Figure S17.1. ^{1}H-NMR spectrum of the compound **12** (500 MHz – CD_3COCD_3)

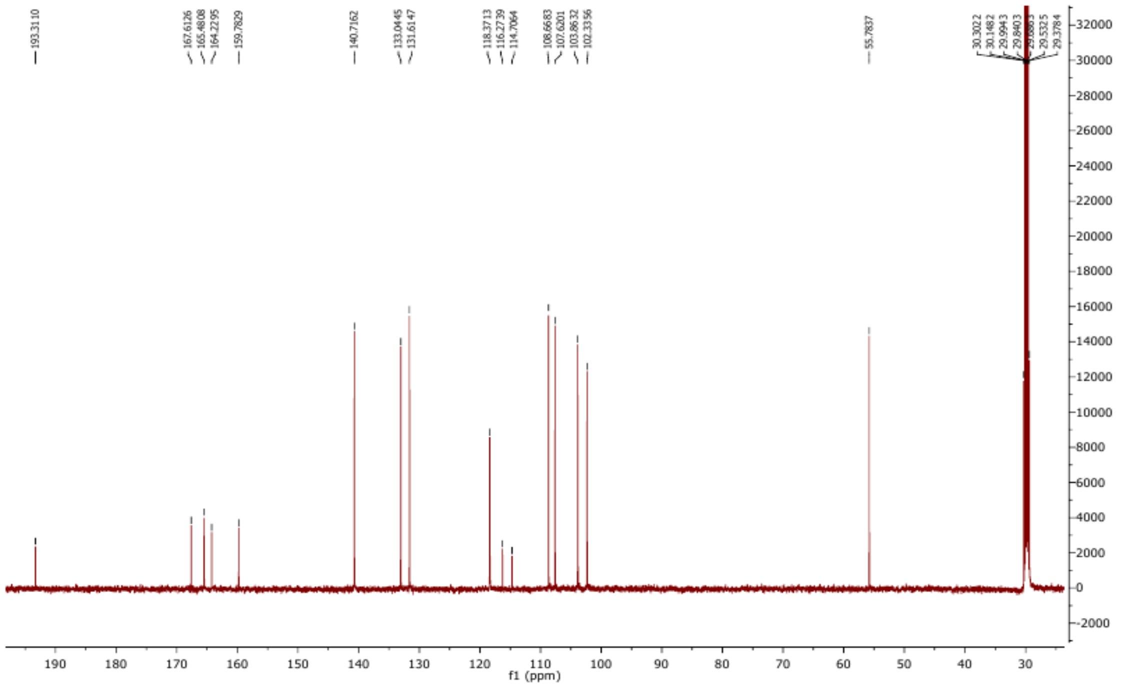

Figure S17.2. ^{13}C-NMR spectrum of the compound **12** (125 MHz – CD_3COCD_3)

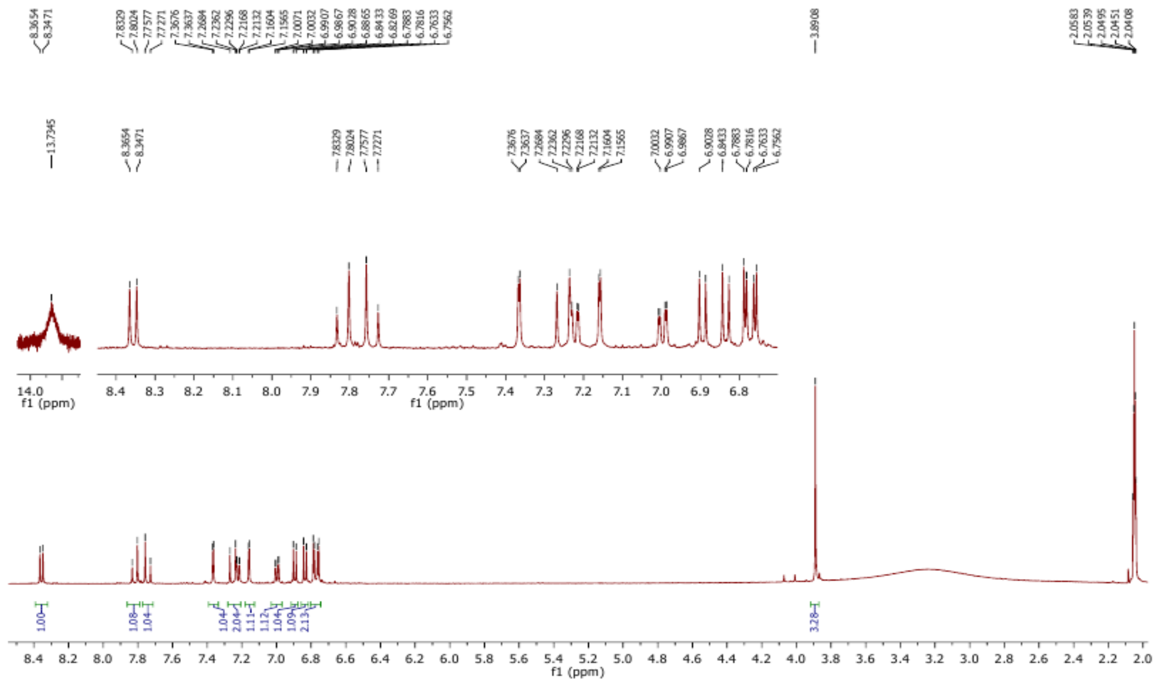

Figure S18.1. ^{1}H-NMR spectrum of the compound **13** (500 MHz – CD_3COCD_3)

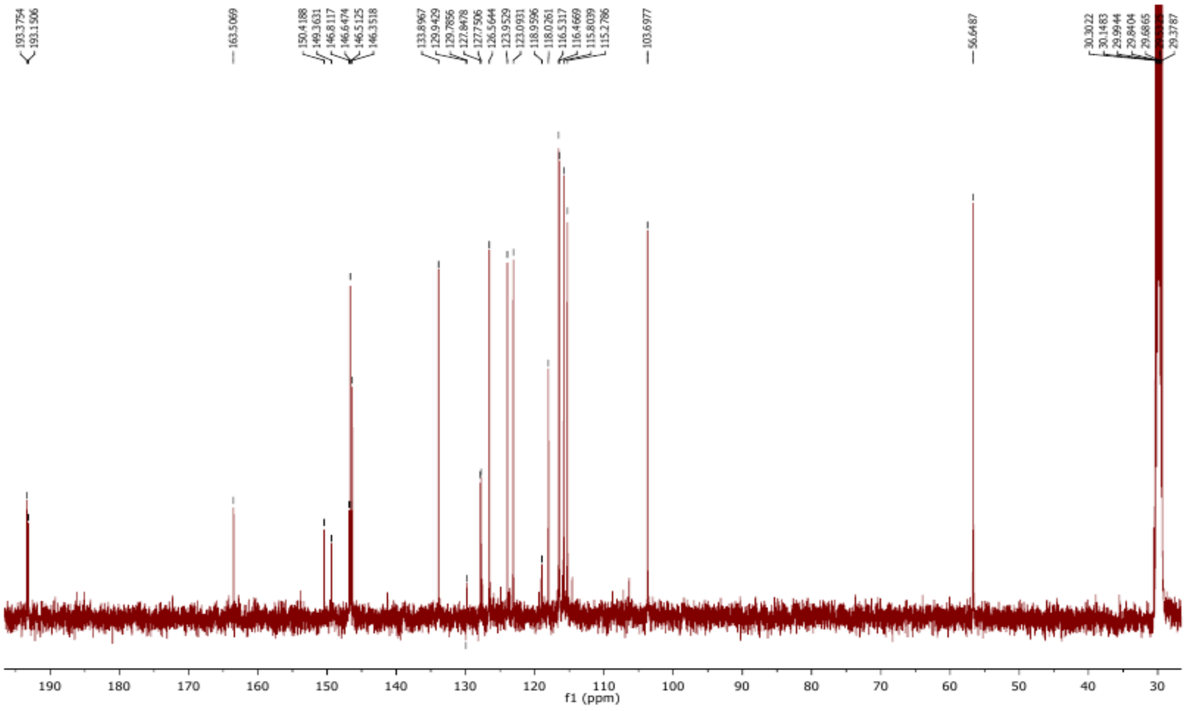

Figure S18.2. ^{13}C-NMR spectrum of the compound **13** (125 MHz – CD_3COCD_3)

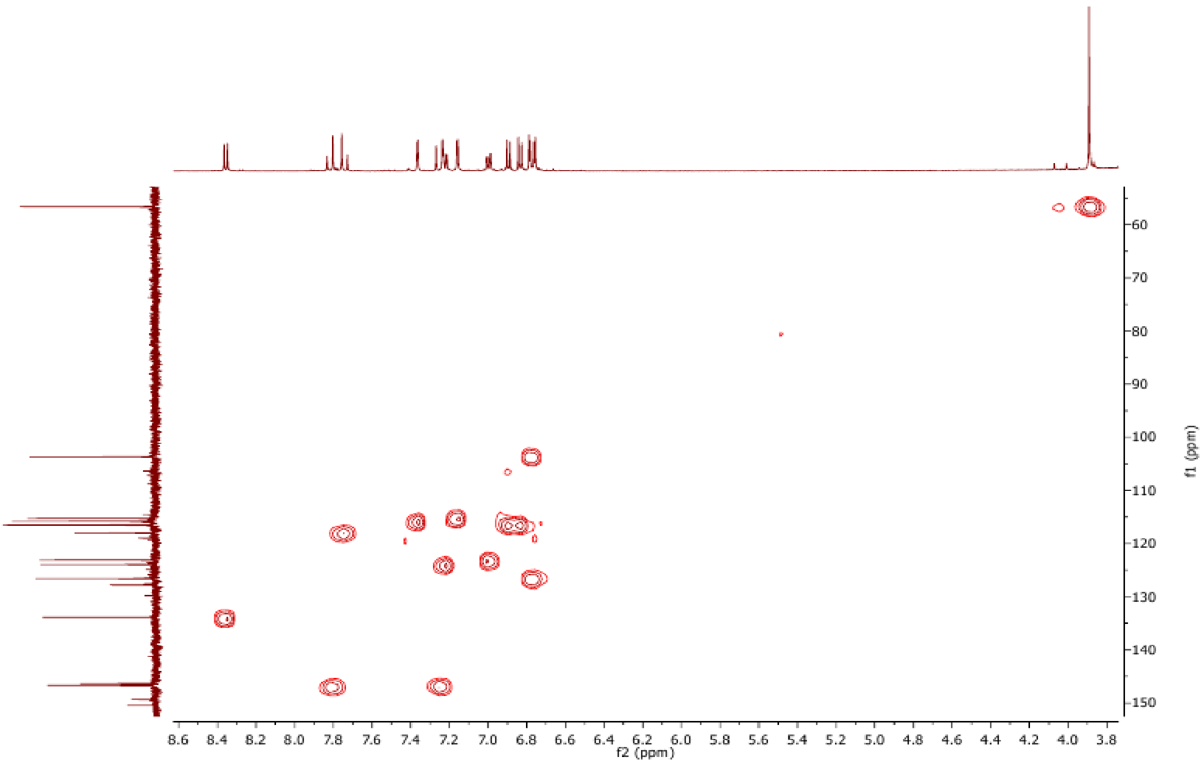

Figure S18.3. HSQC-NMR spectrum of the compound **13**

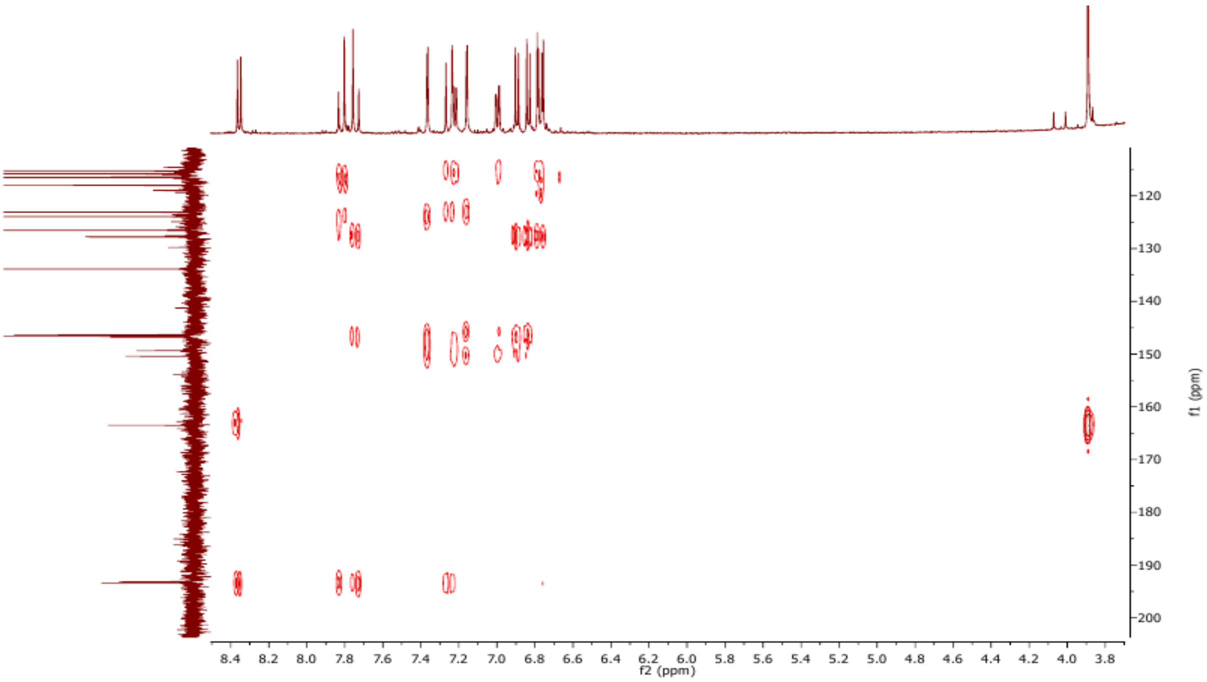

Figure S18.4. HMBC-NMR spectrum of the compound **13**

Generated by Foxit PDF Creator © Foxit Software
http://www.foxitsoftware.com For evaluation only.

Display Report

Analysis Info

Analysis Name	D:\Data\datadmm\mau HC9_1-d,1_01_3282.d	Acquisition Date	2/23/2012 2:36:13 PM
Method	dmm.m	Operator	Mai
Sample Name	mau HC9	Instrument	micrOTOF-Q 10187
Comment			

Acquisition Parameter

Source Type	ESI	Ion Polarity	Negative	Set Nebulizer	1.2 Bar
Focus	Not active	Set Capillary	4500 V	Set Dry Heater	200 °C
Scan Begin	100 m/z	Set End Plate Offset	-500 V	Set Dry Gas	6.0 l/min
Scan End	3000 m/z	Set Collision Cell RF	550.0 Vpp	Set Divert Valve	Source

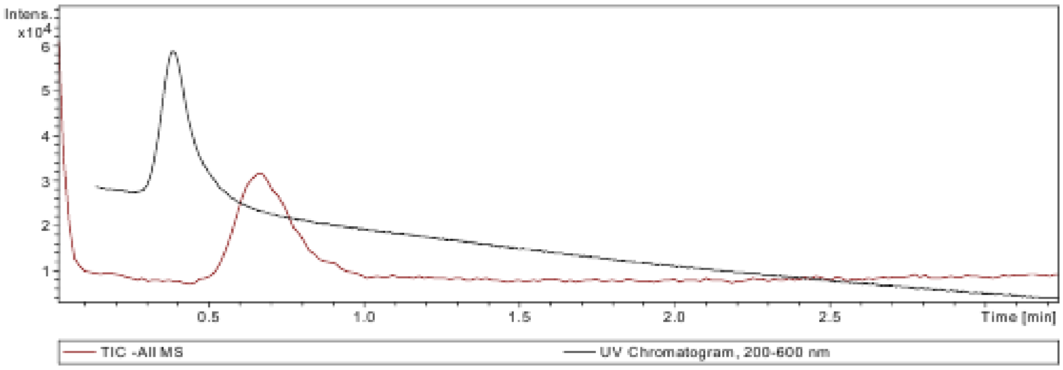

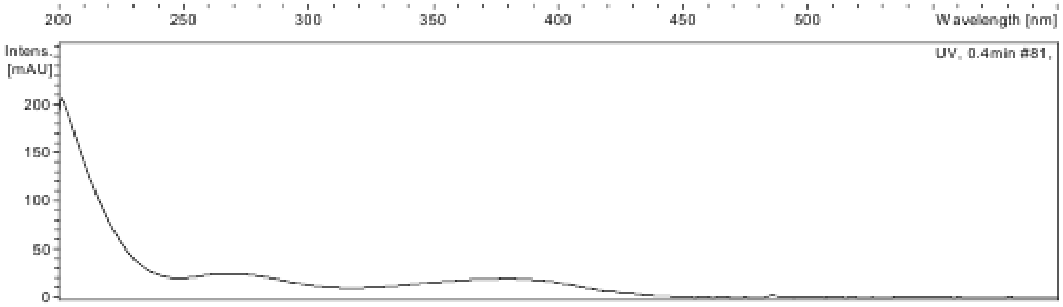

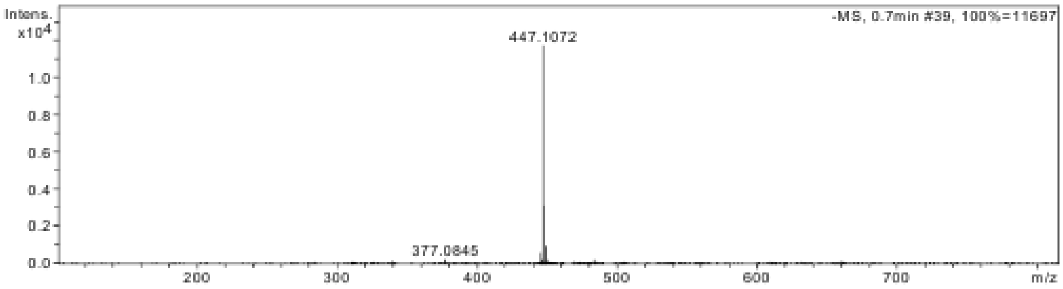

Figure S18.5. HR-ESI-MS spectrum of the compound **13**

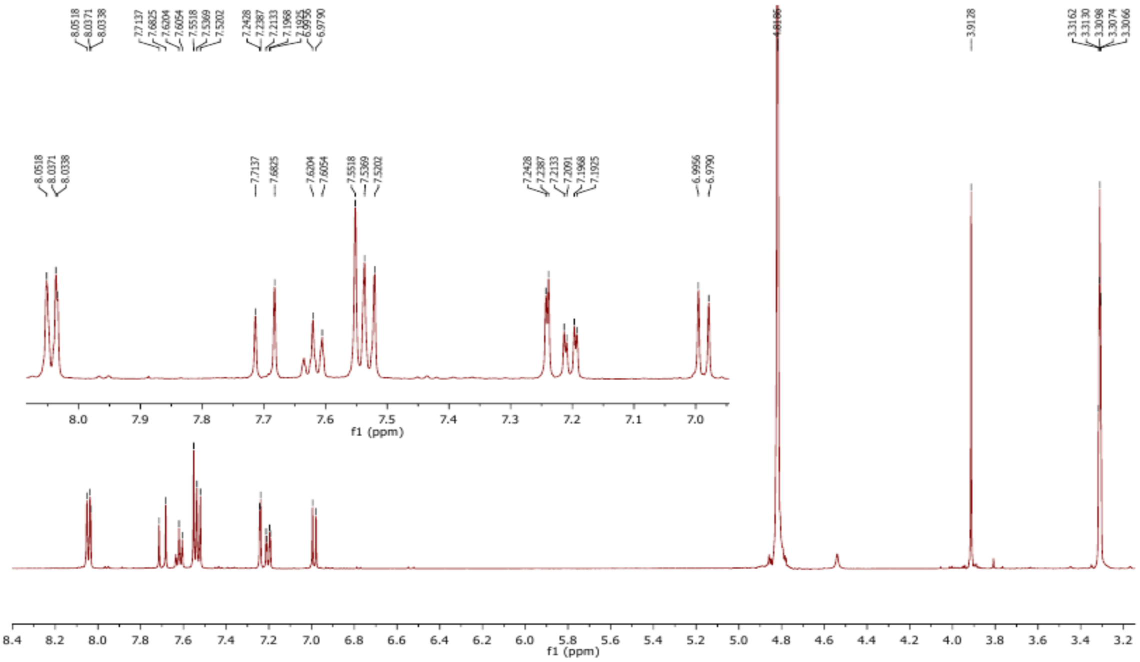

Figure S19.1. ^{1}H-NMR spectrum of the compound **14** (500 MHz – CD_3OD)

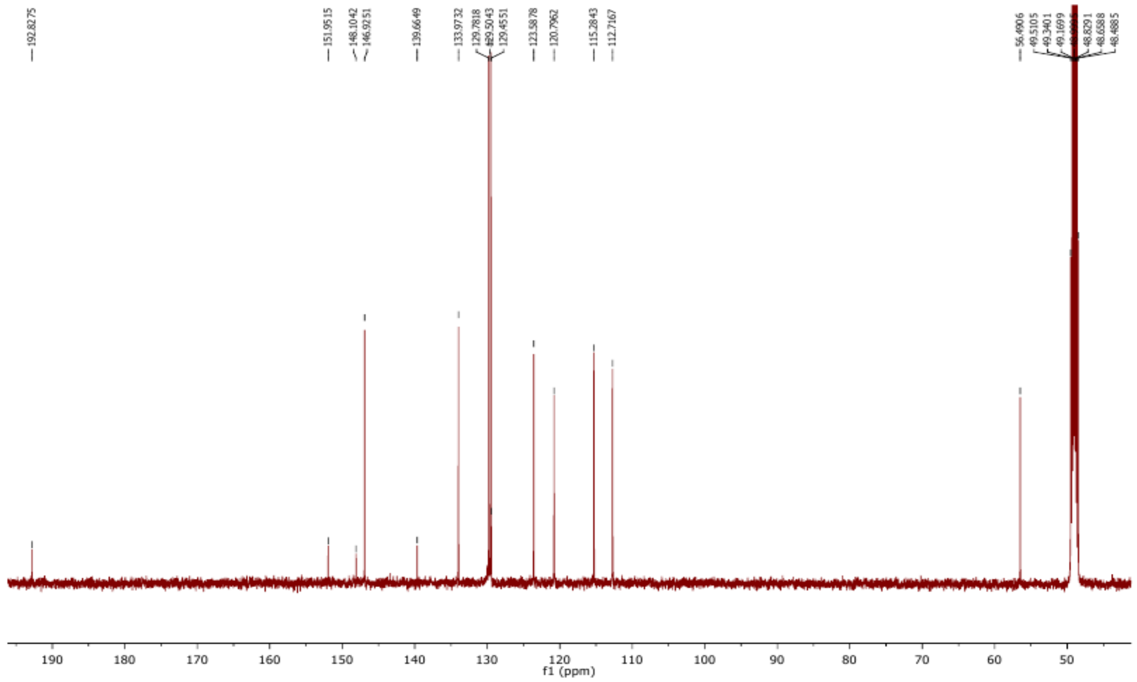

Figure S19.2. ^{13}C-NMR spectrum of the compound **14** (125 MHz – CD_3OD)

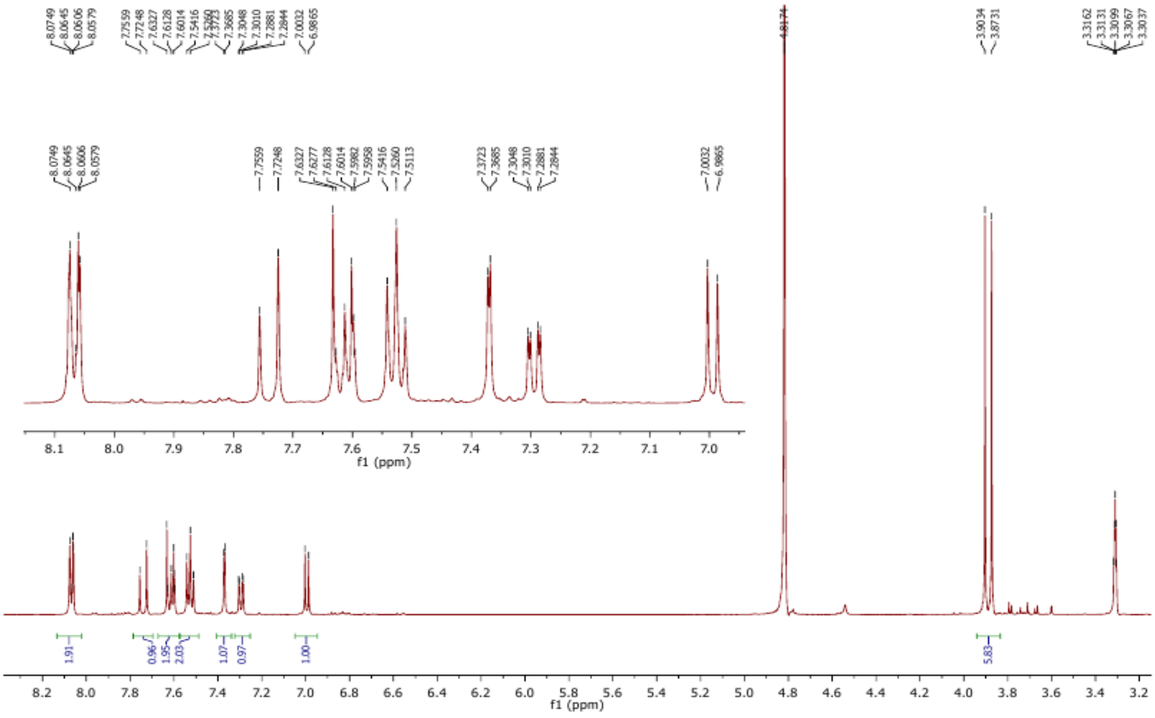

Figure S20.1. ^{1}H-NMR spectrum of the compound **15** (500 MHz – CD_3OD)

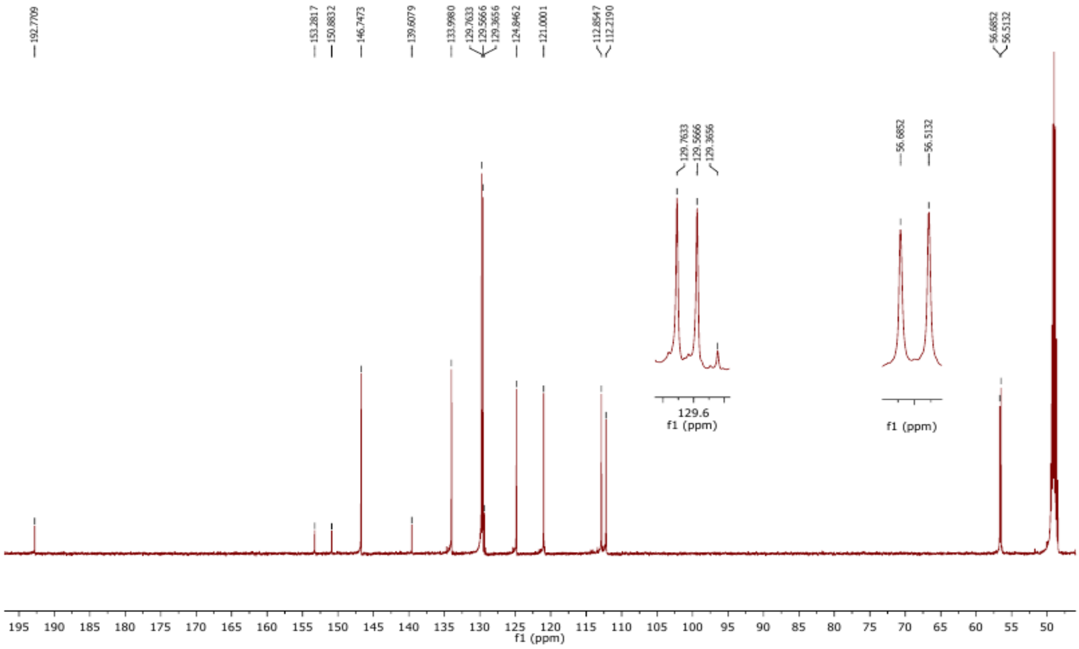

Figure S20.2. ^{13}C-NMR spectrum of the compound **15** (125 MHz – CD_3OD)

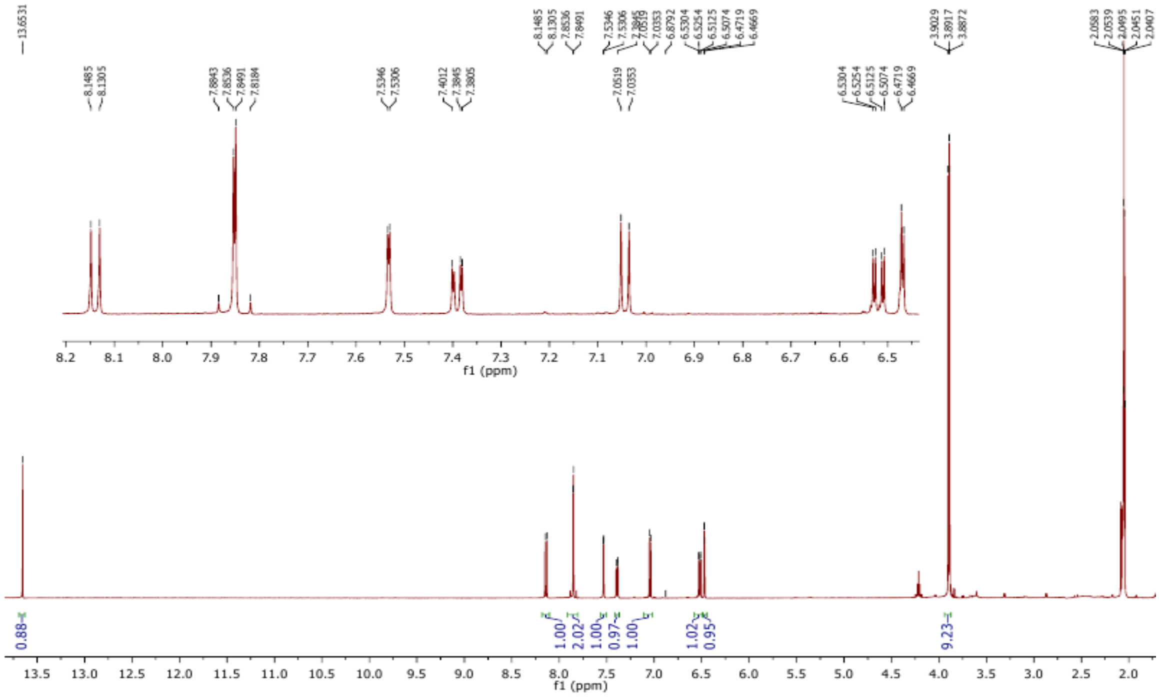

Figure S21.1. ^{1}H-NMR spectrum of the compound **16** (500 MHz – CD_3COCD_3)

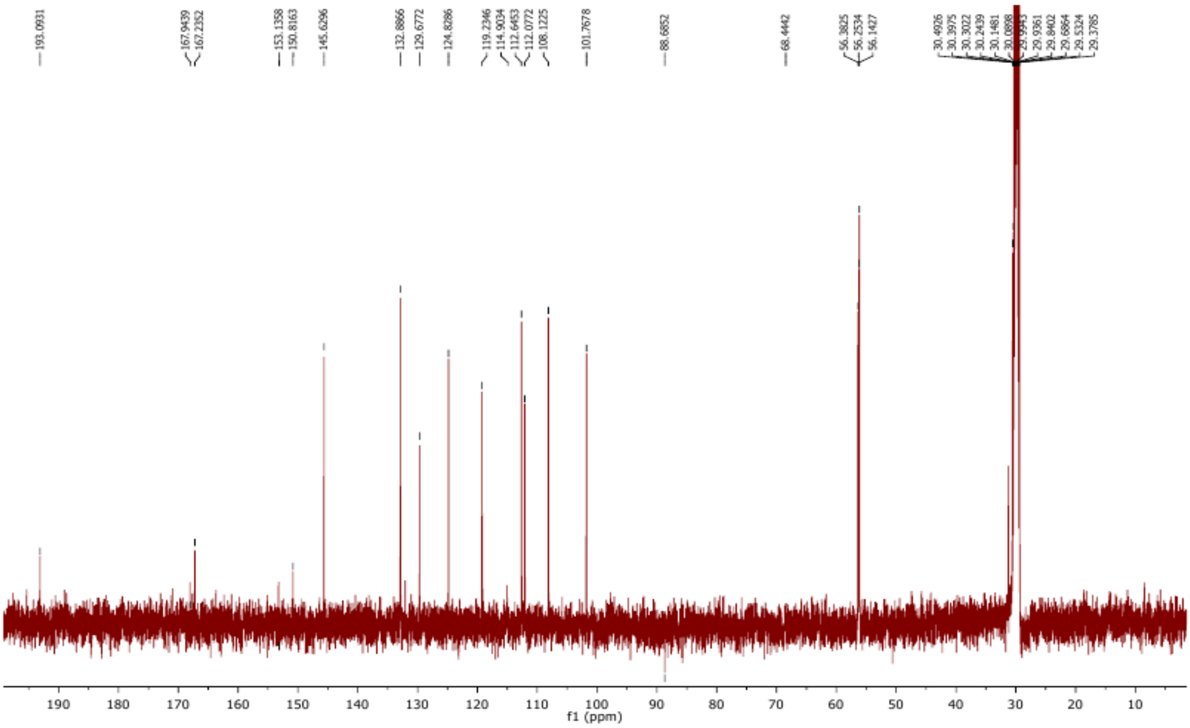

Figure S21.2. ^{13}C-NMR spectrum of the compound **16** (125 MHz – CD_3COCD_3)

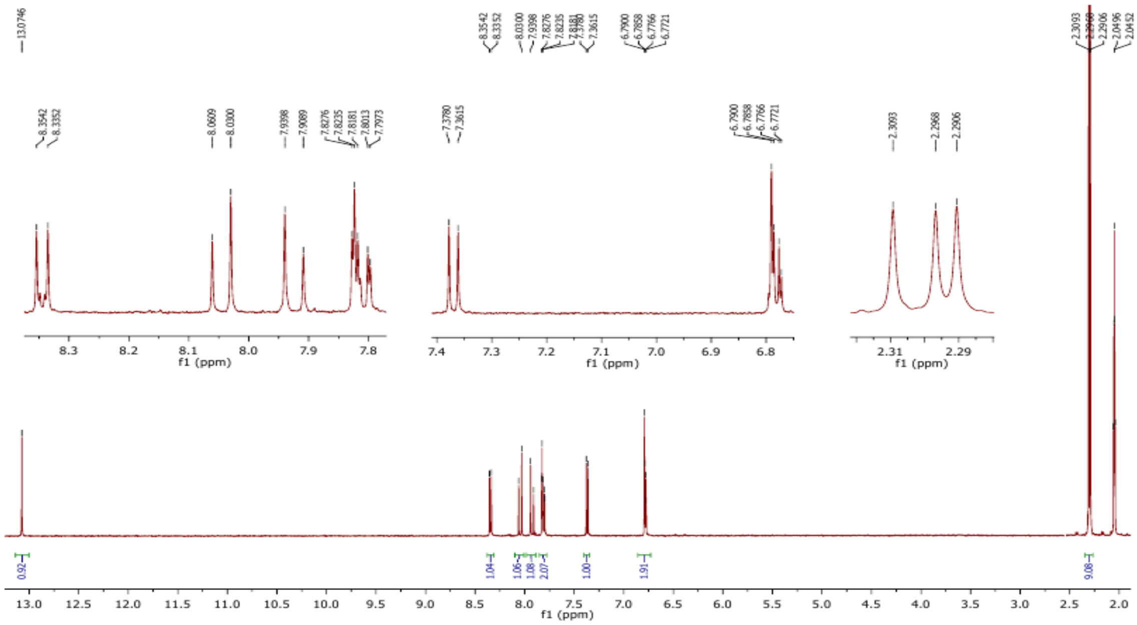

Figure S22.1. ^{1}H-NMR spectrum of the compound **17** (500 MHz – CD_3COCD_3)

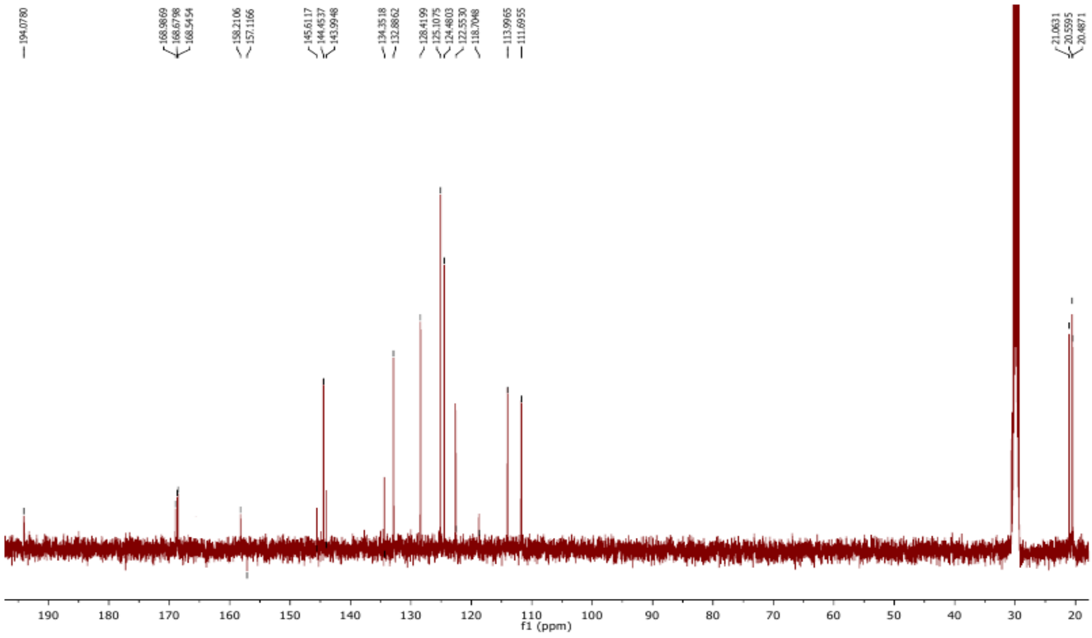

Figure S22.2. ^{13}C-NMR spectrum of the compound **17** (125 MHz – CD_3COCD_3)

Generated by Foxit PDF Creator © Foxit Software
http://www.foxitsoftware.com For evaluation only.

Display Report

Analysis Info

Analysis Name	D:\Data\datadmm\mau HC8_1-d,1_01_3283.d	Acquisition Date	2/23/2012 2:41:58 PM
Method	dmm.m	Operator	Mai
Sample Name	mau HC8	Instrument	micrOTOF-Q 10187
Comment			

Acquisition Parameter

Source Type	ESI	Ion Polarity	Negative	Set Nebulizer	1.2 Bar
Focus	Not active	Set Capillary	4500 V	Set Dry Heater	200 °C
Scan Begin	100 m/z	Set End Plate Offset	-500 V	Set Dry Gas	6.0 l/min
Scan End	3000 m/z	Set Collision Cell RF	550.0 Vpp	Set Divert Valve	Source

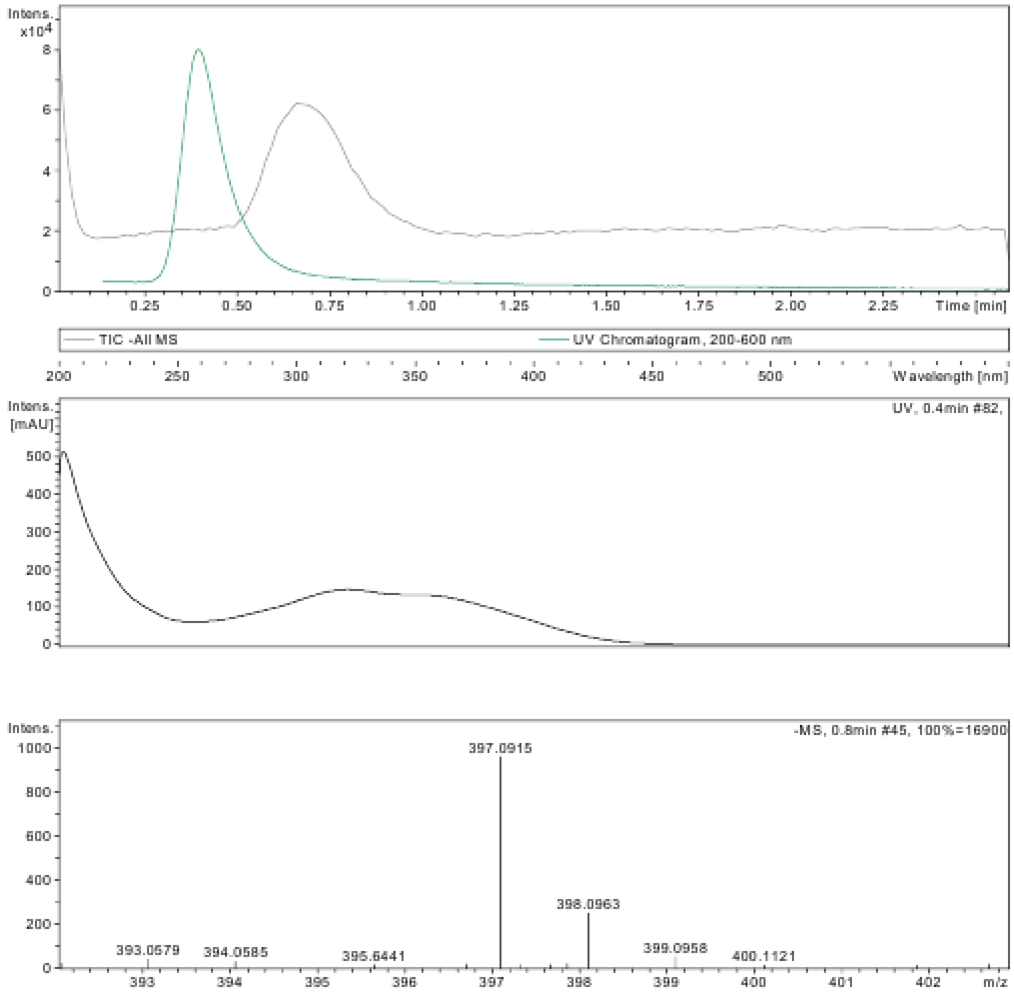

Figure S22.3. HR-ESI-MS spectrum of the compound **17**
